# Purification and structural characterization of the Na^+^-translocating ferredoxin: NAD^+^ reductase (Rnf) complex of *Clostridium tetanomorphum*

**DOI:** 10.1038/s41467-022-34007-z

**Published:** 2022-10-23

**Authors:** Stella Vitt, Simone Prinz, Martin Eisinger, Ulrich Ermler, Wolfgang Buckel

**Affiliations:** 1 Department of Molecular Membrane Biology, Max Planck Institute of Biophysics, Max-von-Laue-Str. 3, Frankfurt am Main, Germany; 2 Philipps-Universität Marburg, Faculty of Biology, Karl-von-Frisch Straße 8, Marburg, Germany; 3 Department of Structural Biology, Max Planck Institute of Biophysics, Max-von-Laue-Str. 3, Frankfurt am Main, Germany

**Keywords:** Cryoelectron microscopy, Enzyme mechanisms, Bioenergetics, Permeation and transport

## Abstract

Various microbial metabolisms use H^+^/Na^+^-translocating ferredoxin:NAD^+^ reductase (Rnf) either to exergonically oxidize reduced ferredoxin by NAD^+^ for generating a transmembrane electrochemical potential or reversely to exploit the latter for producing reduced ferredoxin. For cryo-EM structural analysis, we elaborated a quick four-step purification protocol for the Rnf complex from Clostridium tetanomorphum and integrated the homogeneous and active enzyme into a nanodisc. The obtained 4.27 Å density map largely allows chain tracing and redox cofactor identification complemented by biochemical data from entire Rnf and single subunits RnfB, RnfC and RnfG. On this basis, we postulated an electron transfer route between ferredoxin and NAD via eight [4Fe-4S] clusters, one Fe ion and four flavins crossing the cell membrane twice related to the pathway of NADH:ubiquinone reductase. Redox-coupled Na^+^ translocation is provided by orchestrating Na^+^ uptake/release, electrostatic effects of the assumed membrane-integrated FMN semiquinone anion and accompanied polypeptide rearrangements mediated by different redox steps.

## Abstract (web summary)

Rnf reversibly reduces NAD^+^ by ferredoxin coupled with Na^+^/H^+^ pumping for microbial ion gradient or reduced ferredoxin formation. Here, a cryo-EM structure provides information about the participating iron and flavin cofactors, the electron transfer route and the putative Na^+^ passage.

## Introduction

Redox-driven ion-gradient formation across biological membranes is a pivotal instrument of cellular life to power processes like ATP synthesis, solute symport, and locomotion. Most organisms use the classic respiratory chain primarily consisting of proton-pumping membrane-spanning complex I, bc_1_ complex, and cytochrome *c* oxidase for energy conservation but, in addition, variations and alternative solutions are created during evolution to harness special environmental demands. Anaerobic microorganisms as fermenting, acetogenic, and sulfate-reducing bacteria and methanogenic archaea have developed, very earlier in evolution, ion-translocating machineries that incorporate energy-rich reduced ferredoxin (Fd_red_) carrying redox-active Fe/S clusters (E°′ ~ ‒450 mV) or flavodoxin for electrochemical gradient formation (Supplementary Fig. 1)^1–3^. Electron acceptors are either H^+^ (E°′ = ‒414 mV)^4,5^ or NAD^+^ (E°′ = ‒320 mV)^6^. The latter electrogenic NAD^+^-ferredoxin oxidoreductase reaction is catalyzed by the membrane-spanning multi-subunit complex Rnf (from Rhodobacter N_2_ fixation metabolism, in which context Rnf was discovered^7,8^) thereby linking ferredoxin and pyridine nucleotide pools (Supplementary Fig. 1)^9^. Dependent on the organism, H^+^ and Na^+^ can serve as pumping ion^10^. NAD^+^ reduction by Fd_red_ oxidation proceeds with a driving force of ~ ‒20 kJ/mol, which allows transport of one or maximally two Na^+^/H^+^ across the membrane. Fd_red_ is generated by flavin-based electron bifurcation processes (Supplementary Fig. 1) and diverse exergonic oxidation reactions e.g., those from pyruvate to acetyl-CoA (Supplementary Fig. 1), from CO to CO_2_ and from formate to CO_2_^1^_._ Due to the reversibility of the Rnf reaction, Fd_red_ can be also produced at expense of the transmembrane ion gradient. The strong electron donor Fd_red_ is required to reduce low-energy compounds as N_2_, CO_2_, NO_2_, and the regulatory protein SoxR (activated by Fe/S oxidation as response of oxidative stress) which is impossible with the normally used NADH^11^. The reversed process is also applied by aerobic microorganisms.

Comprehensive research on Rnf has been hampered for a long time by the impossibility to prepare Rnf as pure and homogeneous protein complex^12^, although functional studies in the membrane-bound state and genetic approaches provide compelling evidences for its ferredoxin-NAD^+^ oxidoreductase activity. Ion transport measurements on the acetogenic bacterium *Acetobacterium woodii* and, very recently, on the fermenting bacterium *Thermotoga maritima* demonstrated the oxidation of Fd_red_ by NAD^+^ to be coupled with Na^+^ translocation from the outside into the lumen of inverted membrane vesicles^13–15^. The Na^+^ dependency of the Rnf reaction has been further shown at inverted vesicles of the fermenting bacterium *Acidaminococcus fermentans*^3^. Eliminations of Rnf genes from *A. woodii*^16^, *Methanosarcina acetivorans*^17^*, Bacteroides fragilis*^18^, and *Clostridium thermocellum*^19^ genomes result in phenotypes that are directly correlated with the loss of the membrane-bound ferredoxin-NAD^+^ oxidoreductase activity. According to sequence analysis, RnfA (21 kDa), RnfD (33 kDa), and RnfE (21 kDa) are integral membrane proteins with 5–7 predicted transmembrane (TM) helices whereas RnfB (30 kDa) and RnfG (20 kDa) contain one such helix probably as a membrane anchor. RnfC (47 kDa) is non-covalently attached to the cytoplasmic side of the residual complex^20^. Architecturally, Rnf shows the highest relationship to the electrogenic NADH: ubiquinone reductase complex (Nqr)^11,21^. NqrE shares sequence similarities with RnfA, NqrB (Na^+^ transporting subunit) with RnfD, NqrC with RnfG and NqrD with RnfE. NqrA is slightly related to RnfC and NqrF is unrelated to the Fd-binding subunit RnfB^22^. The Nqr complex, widespread among pathogenic bacteria, couples the reduction of quinone by NADH with the formation of an electrochemical Na^+^ gradient^11,21^. Thus, Rnf operates between ca. ‒500 and ‒300 mV and Nqr between ca. ‒300 and +100 mV^6^. The different redox spans require different electron input and output modules for the related electrogenic enzymes.

In this work, we develop a robust protocol to prepare a pure and homogeneous Rnf complex of the Gram-positive bacterium *Clostridium tetanomorphum* fermenting glutamate to ammonia, CO_2_, acetate, butyrate, and hydrogen (Supplementary Fig. 1). In parallel, an effective purification procedure has been also established for the enzyme from *A. woodii*^23^ and *T. maritima*^14^. Furthermore, we analyze the cofactor content and present the cryo-EM structure of the labile and highly oxygen-sensitive Rnf complex after reconstitution into a nanodisc. Previous studies on enriched and active Rnf of *C. tetanomorphum* have revealed initial information about the molecular composition, cofactor content, and the biochemical reaction^12,24^. During the revision process the Rnf structure of *Azotobacter vinelandii* has been published^25^.

## Results

### Preparation of native *C. tetanomorphum* Rnf and molecular characterization

**Fig. 1:**
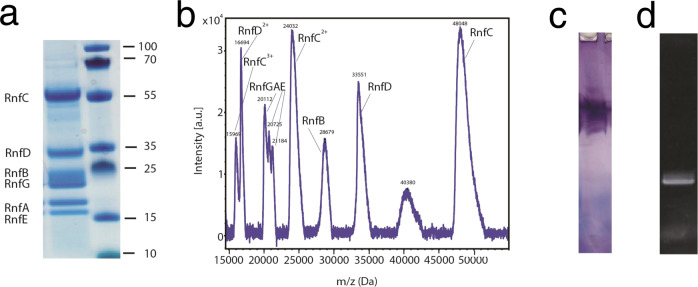
Biochemical analysis of the Rnf complex. **a** SDS-PAGE. The individual bands could be assigned as subunits RnfE, RnfA, RnfG, RnfB, RnfD, and RnfC. Molecular mass markers are given in kDa. The experiment was done twelve times. **b** Mass spectrometry. Subunit identification was performed by MALDI MS analysis. Mass spectra were recorded with Rnf (2 mg/ml) in buffer A by using a MALDI–time-of-flight (TOF)/TOF mass spectrometer. **c** Clear native PAGE (performed thrice). The position and functionality of Rnf become visible after incubating the gel with nitro tetrazolium blue and NADH as an enzymatic assay. **d** SDS-PAGE without Coomassie staining but irradiated with UV-light to identify covalently attached FMN cofactors via a fluorescence band (performed eighteen times). Source data are available as a Source data file.

Previous attempts to prepare Rnf of *C. tetanomorphum* did not lead to a sufficient quality for serious structural and functional studies. Therefore, a completely new protocol was elaborated, composed of four attuned chromatographic steps executed in a time slot of 15 h that accomplishes besides the purification further functions (Supplementary Fig. 2). In the initial hydroxyapatite chromatographic step Rnf was concentrated to increase its stability. The following NADP binding red active agarose affinity column was overloaded with a Rnf-containing protein solution to minimize non-specific binding. Overnight size exclusion chromatography was used to desalt the Rnf sample and to remove aggregation and degradation products. In contrast to other proteins, the final strong anion exchange column did not bind Rnf, which was eluted as a sharp peak, sufficiently concentrated for further use. Thus, Rnf purification proceeded without a conventional concentration step that may have disrupted the multi-subunit complex. Even by minute O_2_ exposure, the enzyme disassembled and the purification procedure had to be stopped. Buffers and solubilization solution were therefore degassed for several days and the columns were equilibrated with the appropriate buffers one day before the planed purification. The yield was ca. 20 mg of homogeneous Rnf (Fig. 1) from routinely used 20 g cells.

The integrity of the entire Rnf complex was estimated by mass spectrometry, size-exclusion chromatography, clear native PAGE, negative-stain EM, and enzymatic assays. SDS-PAGE (Fig. 1a) and MALDI-TOF (Fig. 1b) analysis of the Rnf solution after purification revealed the presence of all six subunits. The profile of the gel filtration step revealed one single and uniform peak with a molecular mass of ca. 250 kDa (Supplementary Fig. 2). Clear native PAGE showed one single but broad band with an estimated molecular mass of 200 ± 30 kDa (Fig. 1c). In the negative-stained EM images, Rnf is visible as monodisperse single protein (Supplementary Fig. 3). Specific activities of purified *C. tetanomorphum* Rnf were measured to 650 ± 70 mU/mg using hexacyanoferrate(III) as electron acceptor for NADH oxidation, which was similar to 431 mU/mg previously reported for partially purified Rnf^24^. The biological relevant NAD^+^ reduction with Fd_red_ was determined with a coupled enzyme assay (Supplementary Fig. 1) resulting in a specific activity of 450 ± 50 mU/mg. Rnf activity, tested in the membrane from diverse organisms, are in the range from ca. 5 to 500 mU/mg^18^. In addition, the integrity and the enzymatic activity of the Rnf complex were substantiated by a native PAGE in-gel functional assay (Fig. 1c)^26^.

### Cofactor analysis of the Rnf complex

Metal analysis of the entire *C. tetanomorphum* Rnf complex indicated an iron content of 28–31 mol Fe/mol Rnf. Separately produced RnfB and RnfC (Supplementary Fig. 4) contained 18–20 mol Fe/mol and 8–10 mol Fe/mol, respectively. On this basis, their iron-sulfur content was estimated to five [4Fe-4S] clusters for RnfB and two [4Fe-4S] clusters for RnfC in agreement with the Fe/S cluster signature motifs of the genome sequence (Supplementary Fig. 5). Most likely, the RnfADEG core contains no Fe/S clusters in line with the related Nqr complex^27^. The single iron ion between RnfA and RnfE (see below) cannot be detected by the resolution of the method. Previous studies on complete Rnf and individual subunits of several organisms revealed a covalently bound FMN in RnfD and RnfG^12,14,23,28,29^ in line with reports on Nqr^30,31^. Using prepared Rnf of *C. tetanomorphum* UV light was quenched at the position of RnfG but not of RnfD on a SDS-PAGE without staining with Coomassie brilliant blue (Fig. 1d). Treatment of denatured Rnf with phosphodiesterase before SDS-PAGE removed the RnfG band, thereby indicating FMN to be attached via a phosphodiester bond to a conserved threonine (Supplementary Fig. 5f) as originally found for the *Vibrio cholerae* enzyme^28^ and for the related Nqr^31^. A covalently bound FMN was also identified in separated RnfG (Supplementary Fig. 4) and a non-covalently bound FMN in separated RnfC (Supplementary Fig. 4). No flavin was detected in separated RnfB. Preliminary HPLC experiments (data not shown) and a previous work^12^ suggested the presence of riboflavin as prosthetic group, which was very recently confirmed^25^.

### Cryo-EM structure determination and overall structure

**Fig. 2:**
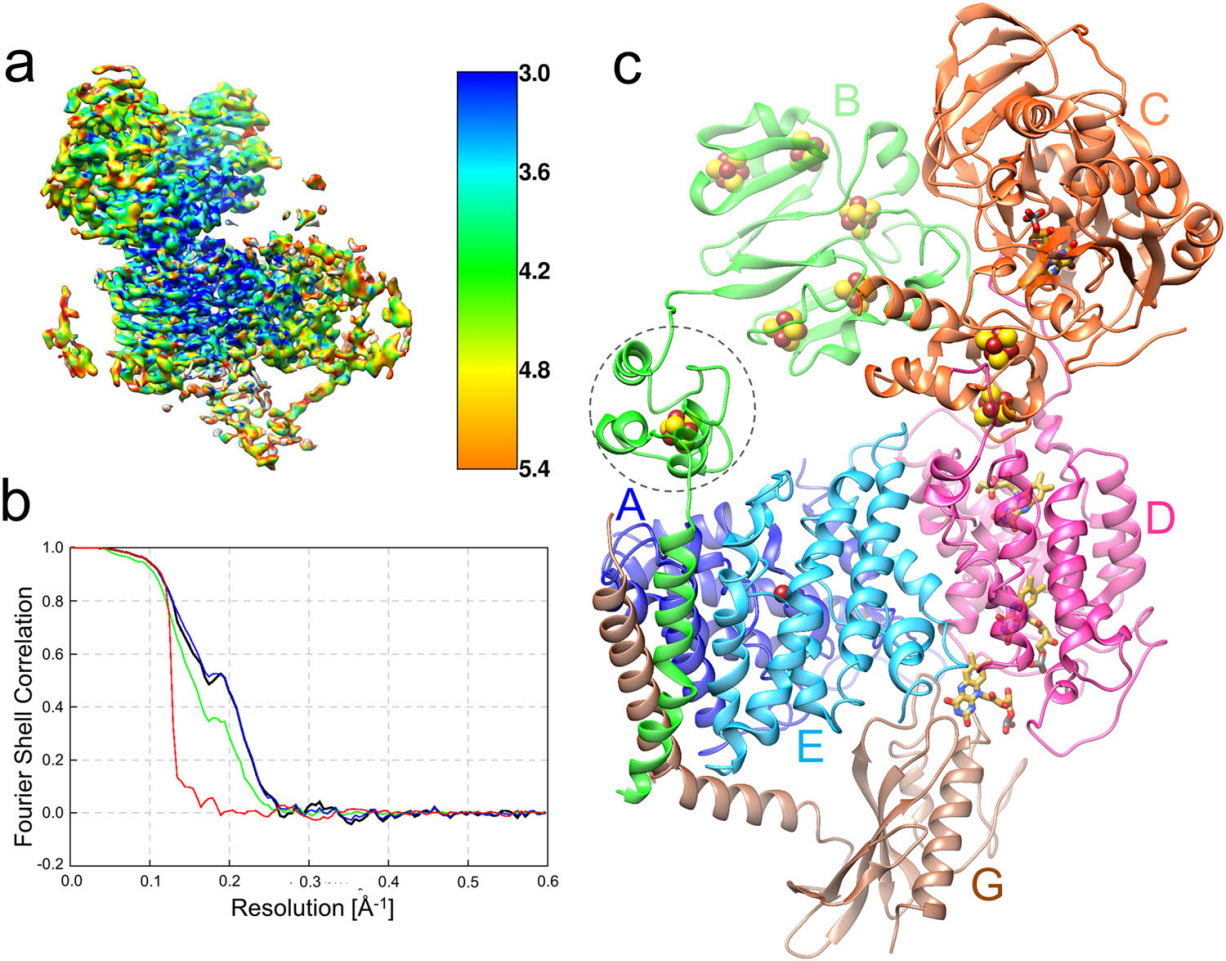
Cryo-EM structure of Rnf from *C. tetanomorphum*. **a** Cryo-EM map of Rnf color-coded according to the local resolution. **b** Gold-standard FSC plot; blue curve is FSC-corrected and green curve is FSC-unmasked. Resolution estimated at FSC = 0.143. **c** Ribbon model. The Rnf or RnfABCDEG structure consists of membrane-spanning RnfADE (blue A, pink D, sky blue E), extracellular RnfG (brown G), and cytoplasmic RnfB (green B) and RnfC (orange C). RnfB and RnfG contain one single TM helix; both form together with one TM helix of RnfA and RnfE a four-helix bundle. The redox cofactors FMN, RBF, Fe^3+^, and [4Fe-4S] clusters are shown as ball-and-stick models. The highly mobile FdI domain (dotted circle) between the larger FdII-III domain and the TM helix of RnfB could only be positioned but not modeled and was therefore replaced by the Alphafold2 model aligned with two vaguely visible helices.

For cryo-EM analysis, the Rnf complex of *C. tetanomorphum* was reconstituted into a DMPC-filled nanodisc built up of the scaffold protein MSP1D1 (Supplementary Fig. 6) and cryo-EM grids were prepared using a home-made manual plunge freezer in an anaerobic tent (95% N_2_, 5% H_2_). From 5379 micrographs collected, 88,423 particles were chosen to calculate a cryo-EM map (Table 1, Supplementary Fig. 7)^32^ with considerable local resolution variations within the protein complex (Fig. 2a). The overall resolution was determined to 4.27 Å (Fig. 2b). Main chain tracing was reasonably possible for RnfA, RnfB (except for residues 31:95), RnfC, RnfD, and RnfE, as many side chains were visible (Supplementary Fig. 8). The highly mobile RnfG could not be entirely modeled on the basis of the experimental map but plausibly completed first by using templates based on NqrC of *V. cholerae* (18% identity, pdb-code: 4p6v-C) as well as RnfG of *T. maritima* (14%, pdb-code: 3dcz) and later on by an Alphafold2 model^33^.

**Table 1:** Cryo-EM data collection, refinement, and validation statistics

Rnf complex	EMDB-14622 PDB 7ZC6
*Data collection and processing*	
Microscope	FEI Titan Krios G3i
Voltage (kV)	300
Camera	Gatan K3 summit
Exposure time (s)	1
Total dose (e^–^/Å^2^)	40
Dose per frame (e^−^/Å^2^)	1.0
Defocus range (μm)	1.2–2.1
Pizel size (Å) (calibrated)	0.837
Magnification (nominal)	105,000×
No. of micrographs	5379
Initial particle number	288,086
Final particle number	88,423
Map resolution (Å)	4.27
FSC threshold Fourier shell correlation	0.143
*Refinement and validation*	
Map-sharpening B factor (Å^2^)	−188.65
**Model composition**	
Chains	7
Protein (Residues)	1577
Ligands (FMN, RBF, [4Fe-4S], Fe^3+^)	3, 1, 8, 1
RMSD bond length (Å)	0.013
RMSD bond angles (°)	1.9
MolProbity score	1.6
Clash score	4.0
**Ramachandran plot (%)**	
Favored	94.63
Allowed	4.52
Outliers	0.45
Rotamer outliers (%)	0.16
**ADP (B-factor) (min/max/mean)**	
Protein	40.1, 161.3, 92.4
Ligand	60.4, 167.6, 91.9

The overall architecture of the Rnf complex (Fig. 2c) is essentially similar to the expected model based on the Nqr structure^27,34^. RnfA, RnfE, and RnfD are integral membrane subunits. RnfG is associated to its extracellular side. Its soluble domain, connected by a helical linker to a remote TM helix, only forms a small interface with RnfD. The ferredoxin subunit RnfB and the NAD oxidoreductase RnfC are attached to the cytoplasmic side of the membrane-integrated RnfADE unit. RnfB also contains one TM helix (1–29) as anchor, which is linked to a larger globular part (97–268) by a highly flexible intermediate segment (35–95) that does not form an interface to other parts of the Rnf complex (Fig. 2c). RnfC (1–435), which is not endowed with a TM helix anchor, and the soluble part of RnfB are firmly associated to each other and fixed to RnfD. Notably, 20 N- and 10 C-terminal residues of RnfD extend into the cytoplasm. Its N-terminal arm is completely buried inside RnfC (partly at the interface to RnfB) and the C-terminal arm is attached to the RnfC surface (Fig. 2c), thereby explaining the location of RnfC in the membrane fraction^20^. This type of fixation is reminiscent to that of NqrA by NqrB^27^. Superposition between Rnf and Nqr reveals identical subunit locations for the conserved RnfADEG (NqrEBDC) core complex; the input modules RnfB or NqrF are localized close to RnfAE and the related output modules RnfC or NqrA close to RnfD (NqrB). Thus, the two NAD oxidoreductase subunits RnfC and NqrF are placed at different sites (Fig. 2c, Supplementary Fig. 9)^27^.

### The Rnf subunits and their cofactors

**Fig. 3:**
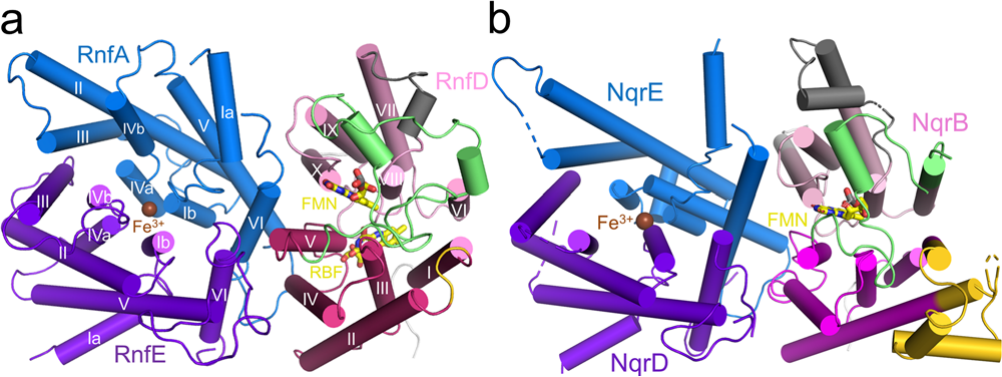
The structure of the integral membrane subunits. **a** RnfADE viewed from the extracellular side onto the membrane. RnfA (sky blue) and RnfE (purple) are inversely arranged and superimposable via a two-fold axis parallel to the viewing direction approximately penetrating the iron. The TM helices are drawn as cylinders. RnfD is built up of two inverse five-helix moieties (red-violet and pink) fused by an expanded linker (lime). The centrally positioned Fe^3+^_RnfAE_ and RBF are located at the cytoplasmic and FMN_RnfD_ at the extracellular monolayer sides, respectively. Helices I and IV of RnfA and RnfE are separated into two half-helices that are displaced and kinked relative to each other. **b** NqrEBD in the orientation of RnfADE. The arrangement and length of the TM helices of RnfADE and NqrEBD are similar. Major differences localized in RnfD/NqrB include the extracellular insertion (gold), the five-helix linker (lime), and the linker (gray) between helices VII (206:223; Rnf nomenclature) and VIII (237:246).

RnfD, the membrane-spanning Na^+^ translocating subunit, is, architecturally, subdivided into N- and C-terminally placed five-helix moieties that are inversely arranged relative to each other (Fig. 3a). This frequently found transporter topology, in particular, resembles that of urea and ammonium transporters^35,36^; the latter also translocates a cation slightly larger than Na^+^. The RnfD structure is most related to that of NqrB^27^ (Fig. 3); the rms deviation between the C_α_ atoms for 304 of 348 residues is 1.7 Å. Deviations include the N- and C-terminal ends, the truncation of 50 extracellularly located residues between helices 20:41 (I) and 47:68 (II) of RnfD localized at the extracellular side, the expanded linker (140–190) between the two five-helix moieties (termed five-helix linker) and the prolonged segment between helices 206:223 (VII) and 237:246 (VIII) (Fig. 3).

**Fig. 4:**
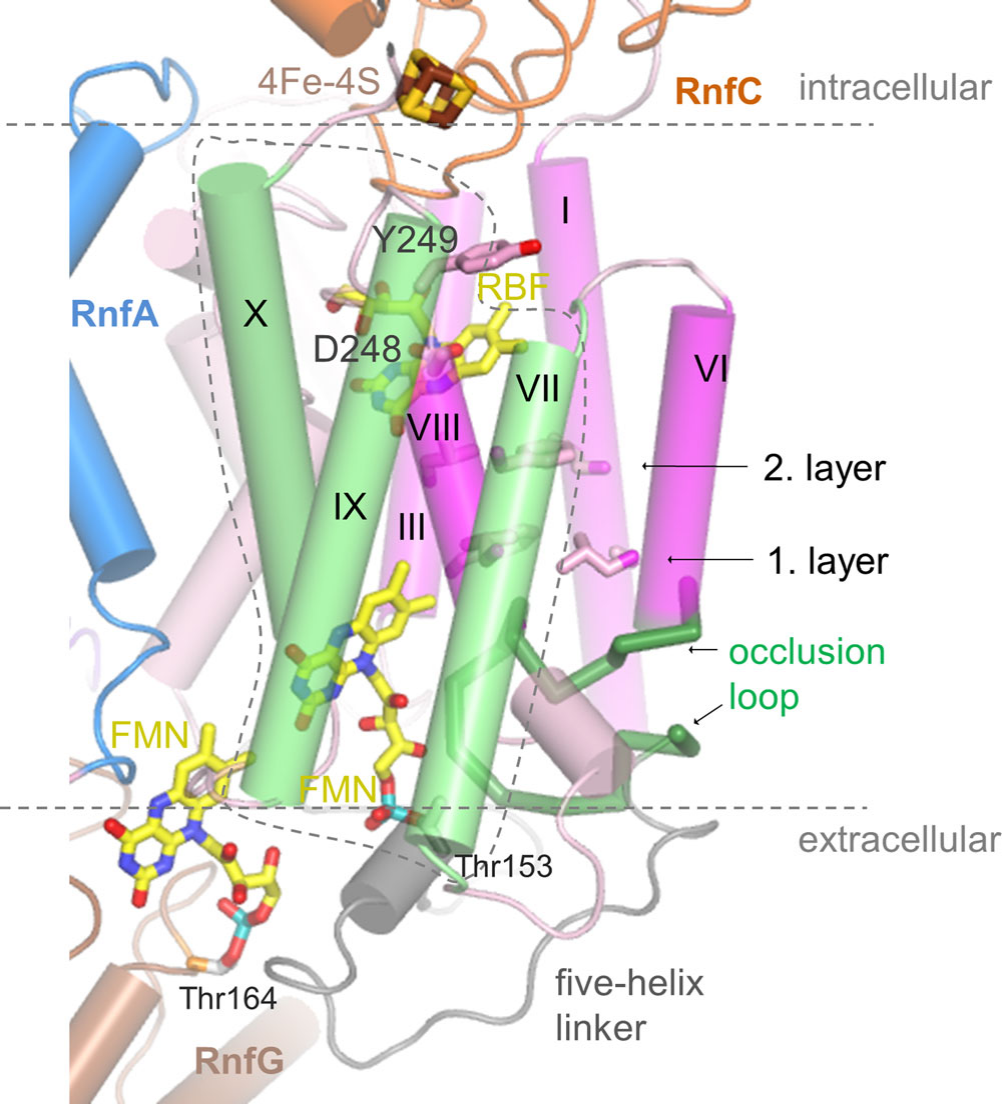
The Na^+^ passage. In the Rnf structure the potential Na^+^ channel is found in a locked state. The constriction between helices I, III, VI, and VIII (magenta) of RnfD subdivided into two hydrophobic layers might be opened during turnover based on findings of architecturally similar transporters; however, alternative passages are possible. The extracellular half-channel is occluded by a loop (dark green) and the cytoplasmic half-channel presumably placed next to Asp248 is rather elusive. FMN_RnfD_ is in ET distance to FMN_RnfG_ and RBF. At the extracellular side, a small helix 230:235 (salmon) arranged nearly parallel to the membrane plane precedes helix VIII (hotpink). The latter contacts the isoalloxazine ring by Gly237. Helices VII, IX, X (green), and VIII (encircled by a dashed line) may move in a concerted fashion to open a passage for Na^+^ transport. The Thr153 and Thr164 hydroxy groups form a covalent bond with the phospho groups of FMN_RnfD_ and FMN_RnfG_, respectively.

As reported for Nqr^27^ RnfD hosts an FMN (termed FMN_RnfD_) embedded in a cavity between the N- or C-terminal ends of TM helices 79:90 (III), 126:136 (V), 237:246 (VIII), and 284:293 (X) that sits in the extracellular lipid monolayer and is covered from the top of the mentioned five-helix linker (Figs. 3a, 4). The phospho group of FMN_RnfD_ is covalently attached to the well-conserved Thr153 hydroxy group (Supplementary Fig. 5d). Its isoalloxazine ring is essentially surrounded by hydrophobic residues except for the moderately conserved Arg130 and Glu185. With its N-terminal end, helix 284:293 (X) points towards the isoalloxazine center and Ser284 (in Nqr exchanged to methionine, Supplementary Fig. 5d) interacts with N5 of FMN_RnfD_ (Fig. 4).

In line with Nqr, related transporters and Rnf sequence analyses, the Na^+^ channel might be localized between central TM helices 20:41 (I), 79:90 (III), 188:201 (VI), and 237:246 (VIII) (Fig. 4). In the structurally characterized resting state, the channel is blocked by two hydrophobic layers. The first layer is primarily formed by Ala35, Phe89, Leu191, and Leu240 and the second by Leu32, Leu86, and Phe244 protruding from the above-mentioned helices. In contrast to the wide vestibule with FMN as part of the side wall in Nqr, the equivalent extracellular half-channel in Rnf is occluded by loop 175–187 protruding in a hairpin-shaped fashion from the solvent-exposed outer wall to the isoalloxazine of the central FMN_RnfD_ (Fig. 4). Thus, Na^+^ cannot bind adjacent to the constriction and the isoalloxazine. In Nqr, the access to the half-channel is substantially modified by the altered five-helix-linker and the mentioned 50 residue insertion (Fig. 3b). The cytoplasmic half-channel of Rnf expands after the hydrophobic barrier to a pocket with a large and unclear density peak (Supplementary Fig. 8a). Due to the availability of the Rnf structure of *A. vinelandii* the density becomes interpretable as riboflavin. Its isoalloxazine ring is flanked by hydrophobic residues except for Asn124 and Asp248. The latter is strictly conserved (Fig. 4, Supplementary Fig. 5) and may mark the Na^+^ binding site. After Asp248 the cytoplasmic vestibule remains narrow (perhaps occluded) although passages of nearly 5 Å to bulk solvent (e.g., between Gly195, Leu198, Ile204, and Ile243) might be sufficient for Na^+^ but not for solvated Na^+^ to escape or enter. In comparison, the cytoplasmic Nqr half-channel is open caused by amino acid exchanges and minor deviations in helix and side chain orientations.

RnfA and RnfE are composed of six TM helices adopting the same fold (rmsd: 1.7 Å; 157 of 187). The two inversely arranged subunits are highly related to their partner subunits NqrE (rmsd 1.6 A; 189 from 191) and NqrD (rmsd 1.7 A; 179 from 194); noticeable overall differences were only detectable at the N-terminal helix and the following loop of RnfA and the short helix 94:101 of RnfE; both changes are close to RnfG. In accordance with Nqr^27^ helices 3:15 + 25:31 as well as 3:15 + 25:32 (I) and helices 104:111 + 114:124 as well as 102:107 + 110:119 (IV) of RnfA and RnfE, respectively, are unwound in their center (Fig. 3). The two half-helices I and IV from both subunits are displaced and kinked relative to each other and the membrane normal resulting in an hourglass-like arrangement and shallow grooves between the four half-helices at the extracellular and cytoplasmic membrane side (Supplementary Fig. 10). One of the non-helical residues at the four kinks is an invariant cysteine (Cys25, Cys113 of RnfA and Cys26, Cys109 of RnfE; Supplementary Fig. 5a, e). The four thiol side chains constitute a metal binding site at the narrowest site of the hourglass (Fig. 3, Supplementary Fig. 8b). As this region is strictly conserved in the Nqr/Rnf families, the metal ion of RnfAE is most likely an iron, which was experimentally identified for Nqr^27^.

**Fig. 5:**
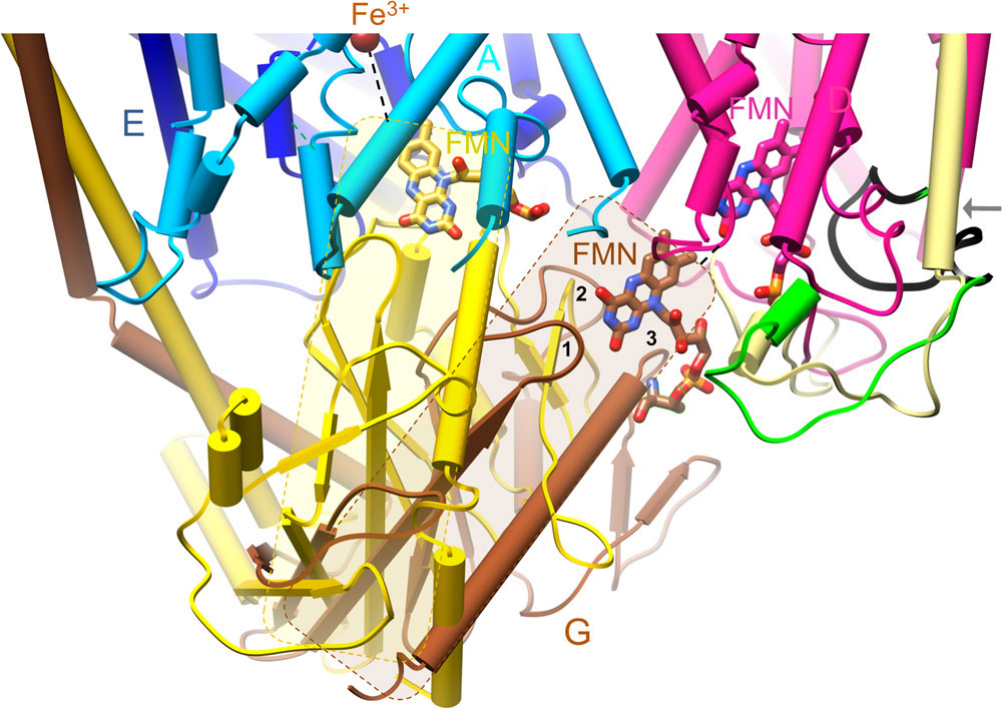
Superimposed RnfADEG and NqrEBDC core structures. RnfG (brown)/NqrC (gold) are primarily built up of a β fold consisting of a central five-stranded antiparallel β-sheet flanked by one long N- and one C-terminal helix on both sides and some elongated linkers between β-strands. The extracellular RnfG is either attached to RnfD (hotpink) as in the cryo-EM structure or to RnfAE (NqrDE) as observed for NqrC in the Nqr crystal structure. Thus, FMN_RnfG/NqrC_ is either in a productive ET distance to FMN_RnfD_ or to the central Fe ion of RnfAE (NqrDE). Mobility of RnfG is rationalized by small respective contact areas. Different orientations between RnfG and NqrC are highlighted by transparent brown and gold rectangles. The exposed covalently attached FMN_RnfG_ is fixed by three protruding loops (marked by 1, 2, 3); the first (91–94) links two β-strands, the second (114–128) a β-strand and a small helical segment parallel to the β-sheet and the third (155–162) precedes the C-terminal helix. The orientation of RnfG substantially influences the conformation of the five-helix linker (green, yellow), in particular, the occlusion loop (black), which is transformed in Nqr to a prolonged helix 188:201 (VI) (gray arrow). The reason for the different orientations of RnfG and NqrC is unknown. Structural differences as e.g., the extracellular insertion 75–124 in NqrB and the deviating orientations of helix 21:42 (I) of RnfD have to be considered but also the high Na^+^ concentration in the buffer (200 mM NaCl) of Rnf and crystallographic effects of Nqr might play a role. Na^+^-dependent conformational changes are reported for NqrC^61^.

Extracellular RnfG basically consists besides the TM helix of a single soluble domain mainly folded as β structure with a long C-terminal α-helix (Fig. 5). This fold is similar to that of NqrC (rmsd = 2.4 Å, 187 of 251)^27^. FMN_RnfG_, attached in an exposed fashion, is held by three partly elongated loops (Fig. 5). In line with the biochemical data, FMN is covalently attached via its phospho group to the conserved Thr164 (Supplementary Fig. 5f) protruding from the N-terminal end of the terminal helix 164:188. Due to the high mobility of RnfG (Fig. 2a), the exact position/orientation of the isoalloxazine ring remains blurred. Most interestingly, RnfG is rotated ca. 35° relative to NqrC^27^, which changes the position of FMN relative to RnfAE and RnfD as well as their redox cofactors (Fig. 5). While NqrC sits in the hourglass-like groove of NqrDE, RnfG is weakly associated with the five-helix linker of RnfD. FMN_RnfG_ points with its xylene group towards FMN_RnfD_, which is completely encapsulated in the presented structure.

**Fig. 6:**
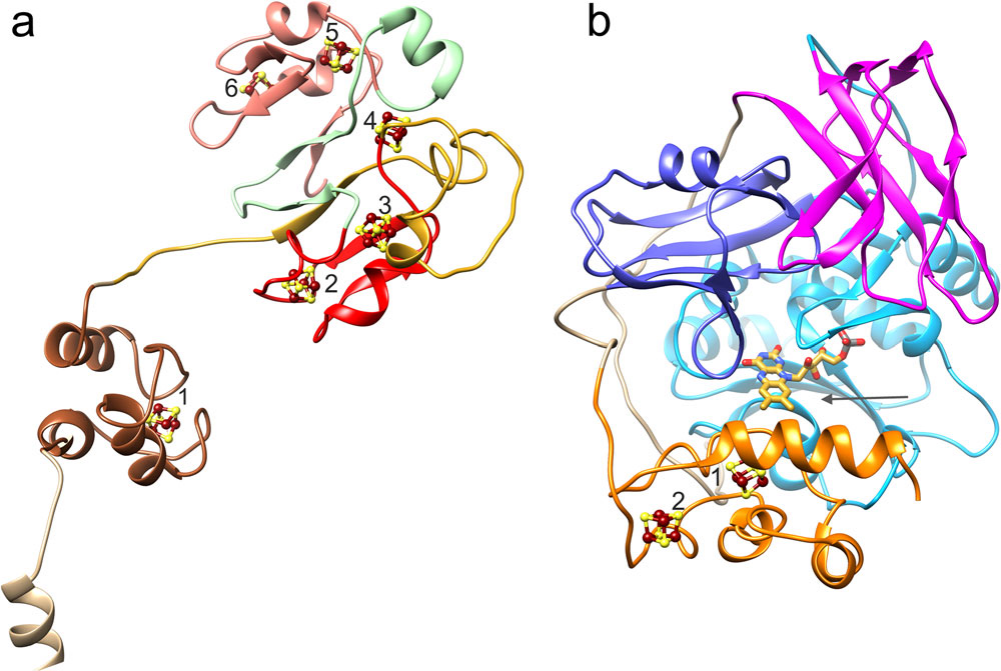
Structure of the cytoplasmic input and output subunits. **a** RnfB. This subunit is built up of the Fd segments FdI (brown), FdII (red), and FdIII (salmon). Each of the latter forming the globular FeII+III domain carries two [4Fe-4S] clusters (Fe and S as brown and yellow spheres). One extra [4Fe-4S] cluster (4) is packed between the segment linking FdII and FdIII (light green) and the RnfC interface segment (gold). The Fd(I) segment is too mobile for reliable model building. Therefore, the FdI structure was calculated by Alphafold2. The position of the [4Fe-4S] clusters was derived from four clustered cysteines but also from a related Fd domain of a corrinoid iron-sulfur protein^37^. **b** RnfC. The subunit is architecturally composed of an N-terminal α,β module built up of three subdomains and a C-terminal Fd domain (orange) carrying two [4Fe-4S] clusters. The ubiquinone-like subdomain (magenta) consists of two three-stranded antiparallel β-strands (30–101) packing against each other and the NuoF-like subdomains (sky blue and blue) of a four-stranded parallel β-sheet surrounded by three helices (102–282) and a five-stranded antiparallel β-sheet (283–352), respectively. The non-covalently bound FMN is embedded at the center of the subunit and accessible for NAD^+^ via a wide cleft (see arrow).

RnfB at the cytoplasmic side is built up besides the N-terminal TM helix of the three soluble Fd-like segments FdI (40–90), FdII (141–189), and FdIII (215–268) (Fig. 6a). FdII and FdIII associated to form the globular FdII+III domain host two [4Fe-4S] clusters each wrapped by the polypeptide and ligated by four cysteines in the canonical manner (Supplementary Fig. 8c). Interestingly, segment 190–214 joining FdII and FdIII as well as segment 91–140 forming the interface with RnfC possess two cysteines each, which constitute a fifth [4Fe-4S] cluster binding site. The five reliably identified [4Fe-4S] clusters are semi-circularly arranged with distances between 9.0 and 10.5 Å to their corresponding neighbors (Fig. 6a). In contrast to the high conservation of other Rnf subunits, the size of RnfB is highly variable among different organisms due to the different number of fused Fd modules (Supplementary Fig. 5b)^14^. The small FdI domain is highly mobile and the density is too noisy for reliable model building and for detecting an iron-sulfur cluster. However, the position of FdI is clearly visible between the N-terminal helix and the FdII+III domain thereby separated from the two fixed anchors by two flexible and completely solvent-exposed linkers (Figs. 2c, 6a). Although the Fe content suggests five [4Fe-4S] clusters per RnfB, a sixth [4Fe-4S] cluster inside FdI is highly likely. First, the shortened *T. maritima* RnfB only consists of the FdI domain^14^ and an absence of any iron-sulfur cluster therein would questionize the general function of RnfB. Second, the Alphafold2 model^33^ reveals a FdI fold with the four cysteines arranged in a manner suitable for accommodating a [4Fe-4S] cluster. A similar Fd fold was also found in the N-terminal domain of a corrinoid and iron-sulfur protein^37^ (rmsd 2.2 Å, 21% sequence identity) also containing a CxxCxxxxxCx_17_C motif for carrying a [4Fe-4S] cluster. Third, the equivalent NqrF also contains a flexible Fd domain carrying a [2Fe-2S] cluster between the N-terminal helix anchor and a globular soluble domain^27^.

Cytoplasmic RnfC is built up of an N-terminal arm (1–30) enveloping the subunit, three compactly associated α, β domains (31–358) and a C-terminally fused Fd domain (359–435) containing two [4Fe-4S] clusters (Fig. 6b). The RnfC structure is related to that of NqrA albeit their high rmsd of 3.9 Å reflects substantial variations of the relative positions of the subdomains and connecting loops. As recognized for Nqr^38^, the second and third α, β domains of RnfC superimpose well with corresponding segments of the NADH binding subunit NuoF of complex I^39^. RnfC hosts a centrally positioned FMN (Supplementary Fig. 8d) that is embedded between the latter two α, β domains and the Fd domain in a related manner as reported for complex I^40^. NqrA devoid of FMN and the two [4Fe-4S] clusters is most likely evolutionary developed from the primordial Rnf complex^22^. The substrate NAD^+^ can reach its binding site in front of the isoalloxazine ring by a wide cleft formed between all domains of RnfC and parts of RnfB (Fig. 6b).

## Discussion

**Fig. 7:**
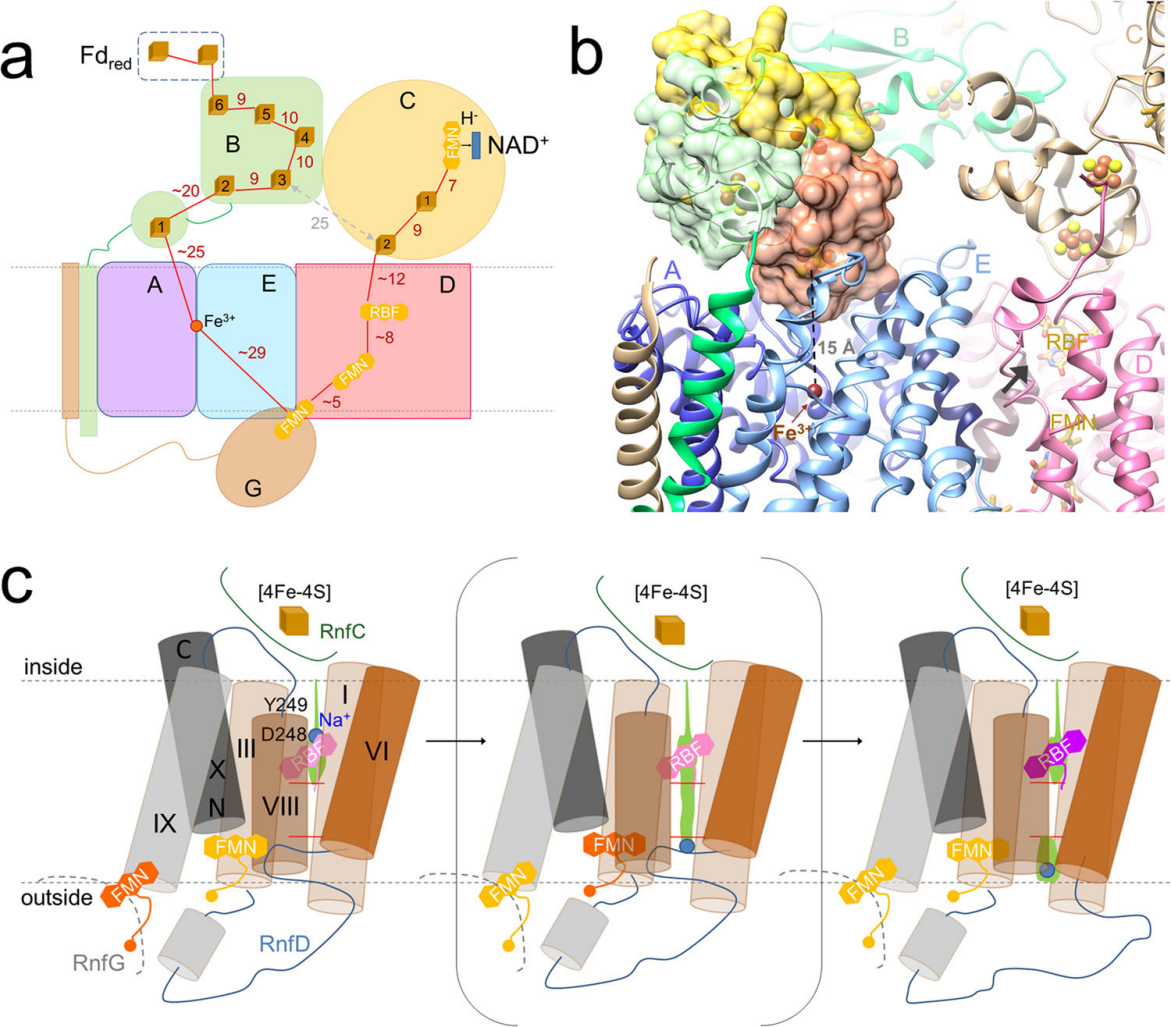
Mechanism of Rnf. **a** Scheme of the ET pathway (highlighted as red lines). The electron flows from the reduced cytoplasmic Fd-like protein (dashed rectangle) modeled in contact with RnfB via RnfB and the membrane-spanning RnfAE to the extracellular RnfG and from there back via RnfD and RnfC to NAD^+^. The [4Fe-4S] clusters are shown as brown cubes and the flavins as yellow triplex-hexagons. The shortest distance between electron carriers of RnfB and RnfC is 25 Å (gray). **b** ET from [4Fe-4S]_RnfB-2_ to Fe_RnfAE_ via Fe_RnfB-1_. FdI (in surface representation) is drawn in green in the position found in the EM structure, in yellow in the position obtained from an Alphafold2 model of RnfB and in coral in the manually modeled position attached to RnfAE. The [4Fe-4S] cluster is shifted ca. 15 Å from the Alphafold2 to the modeled position. The C-terminal helix of RnfAE (dark blue) contacting FdI interacts with helix 126:136 (V) of RnfD (purple) that might be part of the cytoplasmic Na^+^ binding site (indicated by an arrow). **c** Scheme of the redox-driven Na^+^ transport. Na^+^ enter from the cytoplasmic side and binds in front of the locked channel (left panel). Upon channel opening (central panel) Na^+^ migrates to the extracellular half-channel (right panel), from where it is released after conformational change of the occlusion loop. FMN and FMN•^−^ are drawn in yellow and orange, RBFH• and RBFH_2_ in salmon and violet, respectively. The presented scenario is based on conformational changes of helices VII, VIII, IX, and X induced by cytoplasmic Na^+^ binding, RnfG reorientation, low-potential FMN_RnfD_ semiquinone anion formation and accompanied conformational changes. Helices V and VII are omitted for clarity. A switch to turn redox-dependent on and off conformational changes for propagation to an effector site (Na^+^ channel) is a well-known phenomenon in biochemistry^62^.

For structural and mechanistic studies of the Rnf complex of *C. tetanomorphum*, a purification protocol was elaborated that put special emphasis on optimized type and order of chromatographic columns, short purification time, and strict anaerobic treatment. The obtained intact and active enzyme with a rather complete redox cofactor set paves the way to determine a single-particle cryo-EM structure at medium resolution which allows to track the electron transfer (ET) chain between the peripheral iron-sulfur cluster of RnfB and NAD of RnfC and to shed light into the reversible redox-state dependent Na^+^ passage. ET in the direction from Fd_red_ to NAD^+^ is initiated by binding soluble Fd or a Fd-like domain/subunit of a larger protein complex to the RnfB surface in the cytoplasm such that one of its [4Fe-4S] cluster is in a suitable ET distance to the [4Fe-4S]_RnfB-6_ cluster of the FdII+III domain, the one furthest away from the membrane (Figs. 6a and 7a). Subsequently, the electron rapidly flows via [4Fe-4S]_RnfB-5_, [4Fe-4S]_RnfB-4_, [4Fe-4S]_RnfB-3_ to [4Fe-4S]_RnfB-2_. From there the electron is most likely donated to the [4Fe-4S]_RnfB-1_ cluster ~25 Å apart. As the FdII+III domain is kept in its position by multiple interactions with RnfC (Fig. 2c), the highly mobile FdI most likely moves to adjust an ET event. A model for a closer FdI - FdII+III domains arrangement calculated by Alphafold2 resulted in a distance between the corresponding [4Fe-4S] clusters of 8.5 Å (Fig. 7b). The only plausible next ET step that avoids a short circuit proceeds between [4Fe-4S]_RnfB-1_ and Fe_RnfAE_ (Figs. 2c, 7a, b). The gap of 32 Å is predicted to be overcome by swinging FdI by ca. 65° from the FdII+III surface into the groove formed by the four kinked half-helices of RnfAE (Fig. 7b). FdI can be straightforward modeled in a manner that the distance between the two redox cofactors decreases to ca. 15 Å when permitting minor side chain adjustments. The proposed shuttle function of the FdI domain between [4Fe-4S]_RnfB-2_ and Fe^3+^_RnfAE_ corresponds to the role of the NqrF domain carrying the mobile [2Fe-2S] cluster^27^. Worth to note, FdI also interacts with the C-terminal TM helix of RnfA, which contacts helix 126:136 (V) of RnfD, a potential constituent of the cytoplasmic half-channel and the Na^+^ binding site (Fig. 7b). The coupling of Na^+^ uptake and ET from the [2Fe-2S] cluster to FMN, experimentally demonstrated for Nqr, might be structurally realized along this way^41–43^.

According to the redox cofactor arrangement (Fig. 7a), the electron subsequently flows from the Fe_RnfAE_ inside the membrane to extracellular FMN_RnfG_. This ET step is, however, interrupted in the determined structure (Fig. 7a). Transformation from the found blocking state (29 Å) into the conducting state (8 Å) is achieved when RnfG rotates into the position found for NqrC^27^ relative to the fixed membrane-spanning RnfADE/NqrEBD part (Fig. 5). After FMN_RnfG_ reduction and rotation of RnfG into the position of the structurally determined state an electron can be rapidly donated to FMN_RnfD_, the distance between the flavins being <5 Å. This finding confirms the postulated function of RnfG as a shuttle to electronically connect Fe_RnfAE_ and FMN_RnfD_ (Figs. 5 and 7). EPR spectroscopic data on Nqr revealed the one-electron reduced FMN_NqrB_ and FMN_NqrC_ states as anionic semiquinones^43,44^; no data exist for Rnf. Inside the membrane the electron flows from FMN_RnfD_ to riboflavin (7.7 Å) while Na^+^ migrates into the opposite direction (see next paragraph). After ET from riboflavin to the cytoplasmic [4Fe-4S]_RnfC-2_ cluster (11.7 Å) and from there to the neighbored [4Fe-4S]_RnfC-1_ the electron flows to FMN_RnfC_ operating as a 1e-to-2e converter. NAD^+^ can reach the frontside of the isoalloxazine ring by a wide cleft (Fig. 6b) and after a second ET round, a hydride is transferred from the N5H group of reduced FMN_RnfC_ to the nicotinamide C4 of NAD^+^ as e.g., characterized in complex I^40^.

The cascade of events for the redox reaction driven Na^+^ translocation or, in reverse, for the ion gradient consuming Fd reduction remains enigmatic, although the presented data add some pieces to the puzzle (Fig. 7c). Redox-gated Na^+^ pumping might be initiated during binding of the Fd(I) domain to the RnfAE surface resulting in cytoplasmic vestibule widening and Na^+^ uptake and continued upon rotation of RnfG carrying semi-reduced FMN towards RnfD (Fig. 5). As a consequence, the five-helix linker of RnfD undergoes conformational changes, the extracellular and perhaps also the cytoplasmic half-channel becomes occluded and FMN_RnfD_ completely encapsulated (Fig. 7c). The concomitant reduction of FMN_RnfD_ to the postulated semiquinone anion might trigger significant rearrangements of the TM helices perhaps initiated by a response of helix 284:293 (X) (acting as redox sensor) due to the changed interaction between Ser284 at its positively charged N-terminal end and the N5 of the negatively charged isoalloxazine. As a consequence, the neighbored helix 237:246 (VIII), already disengaged upon the cytoplasmic Na^+^ binding, might be displaced outwards (Fig. 7c) as visible in the open-channel structures of the urea and ammonia transporters^35,36^ in agreement with the postulated scenario for Nqr^27^. In the thereby created strained open-channel conformation the postulated Na^+^ binding site next to the highly conserved Asp248 and Asn124 side chains and further main carbonyl oxygens is abolished and Na^+^ passes the widened constriction and reaches after ca. 7 Å a site characterized by the Asn90 and Glu185 side chains attracted by the FMN_RnfD_ semiquinone anion. Concomitantly, the latter conducts an electron to riboflavin rapidly passed on to [4Fe-4S]_RnfC-2_ thereby contributing to the conversion of the strained open-channel into the structurally established relaxed closed-channel state (Fig. 7c). A crucial role is attributed to Asp248 that appears to be involved not only in Na^+^ and riboflavin binding but also in protonation/deprotonation and in adjusting the redox potential of the riboflavin isoalloxazine ring. Upon rotation of RnfG from RnfD to RnfAE the periplasmic hairpin-shaped occlusion loop might be converted into a prolongation of helix 188:201 (VI) (as seen in Nqr, Fig. 5) thereby opening the half-channel and Na^+^ is released into the extracellular space. The reverse ET from NADH to Fd_ox_ is outlined in Supplementary Fig. 11.

Redox-gated ion pumps Rnf and Nqr are unique in biology due to the key roles attributed to flavins not only as 1e-to-2e converter or 1e redox carrier but, most remarkable, also as redox signaling and coupling agent^11,38^. The structural data support the proposed indirect energy coupling implicating that the redox energy is transformed into conformational energy (from a relaxed closed-channel into a strained open-channel state) and then into a Na^+^ gradient or vice versa. However, they do not offer a more detailed mechanistic scenario for coupling rapid downhill ET and slower uphill Na^+^ transfer. In contrast to other redox pumps, where the slower redox reaction inside the membrane is linked with ion translocation, the Fd and NAD redox reactions of Rnf are outside the membrane at the end of the ET chain and not available as coupling sites. As the structurally conceivable ET from FMN_RnfD_ to riboflavin uncoupled with ion translocation has to be prevented, the Na^+^ passage process should be already in a far advanced state before the second ET across the membrane. As already pointed out^11,38^, redox-gated Na^+^ translocation in Rnf/Nqr is a synergetic process of several ET steps coupled with the uptake/release of Na^+^ in the extracellular or cytoplasmic half-channels and the thereby polypeptide rearrangement processes.

## Methods

### Cultivation of *C. tetanomorphum*

*C. tetanomorphum* (DSM 576) cells were regularly grown in a 200 L fermenter in a medium containing 80 mM sodium l-glutamate and 0.6% yeast extract supplemented with a trace element mix (SL10), harvested under anaerobic conditions in the late exponential growth phase and stored at −80 °C^45^.

### Purification of native *C. tetanomorphum* Rnf and molecular characterization

Rnf was purified in an anaerobic tent filled up with a 95%/5% N_2_/H_2_ mixture. For producing 20 mg of protein, 20 g wet cells were suspended in 25 ml lysis buffer (50 mM Tris, pH 7.5 and 20 pM flavin mixture (riboflavin, FAD, and FMN = 1:1:1) and passed four times through a French pressure cell at 125 MPa. Cell debris were removed by centrifugation at 10,000 × *g* and 4 °C for 20 min. After centrifugation at 190,000 × *g* and 4 °C for 1.5 h and washing with lysis buffer the collected membranes were solubilized with 2% 2-dodecyl β-D-maltoside (DDM) and 200 mM NaCl in buffer A (50 mM Tris pH 7.5, 20 pM flavin mixture, 0.05% DDM) with protein concentrations of 2 mg/ml at room temperature for 1 h. Membrane particles were removed at 190,000 × *g* and 4 °C for 30 min.

The solubilized enzymes were loaded under low flow onto a hydroxyapatite column and eluted with a linear phosphate gradient (0–500 mM phosphate) in buffer A. Next, a Reactive Red 120-agarose column (Sigma-Aldrich, Missouri/USA) was used from which the added Rnf-containing fractions were eluted with 2 M NaCl. Finally, the pooled Rnf fractions were applied to a Superdex-200 column (Hiload® 16/600 Superdex 200® pg, Sigma-Aldrich, Missouri/USA), eluted after 57-62 ml, and then to a Poros anion exchange resin column (GoPure™ XQ, 1.2 × 20 cm, Thermoscientific, Massachusetts/USA), eluted with a NaCl gradient (0–1 M NaCl) in buffer A. A source data file is provided for Fig. 1.

For quality control, mass spectra were acquired on a MALDI–time-of-flight (TOF)/TOF mass spectrometer (Bruker Autoflex III Smartbeam) in positive mode (10,000 to 55,000 *m*/*z*) and evaluated using the software Bruker FlexAnalysis 3.3 (Build 75). Beforehand, Rnf (2 mg/ml) was prepared by mixing with acetonitrile (ACN) to promote complex disassembly and afterwards codirectly mixed in a 1:1 ratio with 2,5-dihydroxybenzoic acid (DHB) matrix and applied onto a ground steel MALDI target. Clear native PAGE 4–16% Bis-Tris gels (Invitrogen, Thermo Fisher, Waltham, Massachusetts, USA) were run at 4 °C in the anaerobic tent with a cathode buffer supplemented with 0.05% DDM. Anode buffer, sample buffer and running conditions were performed as specified in the manual^26^. Production and purification of the isolated RnfC, RnfB, and RnfG are described in the legend of Supplementary Fig. 4.

### Determination of the metal and flavin content of *C. tetanomorphum* Rnf

For iron determination protein-bound iron was extracted by acid, Fe^3+^ is reduced to Fe^2+^ with ascorbic acid and complexed with ferene^46^. The absorption of the thereby produced blue dye at 593 nm is proportional to the iron concentration. Covalently bound flavins were detected by exposing SDS-PAGE gel with UV light before Coomassie staining to monitor the quenching of the background fluorescence^28^.

### Functional analysis

Native PAGE in-gel functional assay with nitro tetrazolium blue and NADH were anaerobically prepared as described^26^. A clear blue-colored band served as signal for enzymatic turnover. Rnf activity was anaerobically measured in the assay buffer composed of 150 mM NaCl, 50 mM potassium phosphate, pH 7.5, 20 pM riboflavin, FMN and FAD, 0.05% DDM, 200 µM NADH, and 100 µM hexacyanoferrate (III) in a total volume of 0.5 mL at room temperature. After enzyme addition, the amount of the consumed hexacyanoferrate (III) (*ε*_420_ = 1.02 mM^−1^ cm^−1^) was followed by the decreasing absorbance at 420 nm. In addition, a coupled enzyme assay (see Supplementary Fig. 1) was applied containing 2 μM Rnf, 50 µM Fd_ox_, 250 µM NADH, and 1 µM electron transferring flavoprotein/butyryl-CoA dehydrogenase from *Clostridium difficile* in total volume of 0.5 mL at room temperature^3,47^. The reaction was started with 100 µM crotonyl-CoA and the decrease of the NADH concentration was monitored at 340 nm (*ε*_340_ = 6.3 mM^−1^ cm^−1^).

### Electron microscopy

Negative-stain electron microscopy was initially performed for a basic quality control (Supplementary Fig. 3). For cryo-EM structural analysis, Rnf was extracted from the protein-detergent complex into a nanodisc-lipid device. We used the nanodisc MSP1D1 (49 kDa) forming a diameter of 9.7–12.9 nm and a thickness of 4.5–5.6 nm filled with 120–160 molecules of a lipid. 1,2-dimyistoyl-sn-glycero-3-phosphocholine (DMPC) (Avanti Polar Lipids) turned out to be optimal according to the ratio of aggregates, Rnf-MSP1D1 and empty MSP1D1 (Supplementary Fig. 6A) and negative-stain EM image pattern (Supplementary Fig. 3). MSP1D1 was heterologously overproduced as described^48^ to avoid EDTA in the solution and was made anaerobic before storage at −80 °C. Preparation for cryo-EM with C flat 2/1-300 mesh copper grid (Protochips) was performed in the anaerobic tent using a blotting and freezing procedure with a manual plunge freezer. Briefly, anaerobic Vitrobot’s styrofoam and grids were shortly taken out of the tent the latter being glow discharged for 90 s at 15 mA (Pelco glow discharger). A small cup of ethane was placed into the previously filled liquid N_2_ styrofoam box. After transfer into the humidified anaerobic tent (heated water with wet sponge) the grids were loaded with Rnf sample, blotted for 11 s–13 s only from one side and put into liquid ethane. Movies were recorded using a FEI Titan Krios G3i microscope operated at 300 kV (Thermo Fisher Scientific, formerly FEI Company) and equipped with a Gatan K3 Summit electron detector (Gatan Inc.) and an energy filter of 30 eV slit width. Data collection parameters are given in Table 1. Data were processed with RELION-3.1^49^. Beam-induced motion was corrected using MOTIONCOR2^50^. Initial CTF parameters for each movie were estimated by the Gctf algorithms^51^. Particles were picked with crYOLO^52^ by using manually chosen particles as templates and the dataset was cleaned via 2D classification in RELION-3.1. Unsupervised initial model building, 3D classification, 3D refinement, along with post-processing steps like CTF refinement, Bayesian polishing, and final map reconstructions were performed with RELION-3.1^32,53^. Maps were visualized with Chimera^54^ and models created from Nqr^27^ or Fd^55^ by SwissProt^56^ or manually within COOT^57^. Further real-space refinement was performed using PHENIX^58^ and the quality of the model was assessed with COOT and MolProbity^59^. Finally, the model was reassessed with those from Alphafold2^33^. Structural alignments were performed with COOT and DALI^60^. Figs. 2a, c, 5, 6 and 7b as well as Supplementary Figs. 6b, 8–11 were produced with Chimera, Figs. 3 and 4 with Pymol (Schrödinger, LLC).

### Reporting summary

Further information on research design is available in the Nature Research Reporting Summary linked to this article.

## Supplementary information

Supplementary Information

Reporting Summary

## Data Availability

The data that support this study are available from the corresponding authors upon reasonable request. The cryo-EM maps have been deposited in the Electron Microscopy Data Bank (EMDB) under accession code EMDB-14622 (Rnf complex). The coordinates have been in the RCSB Protein Data Bank (PDB) under accession code 7ZC6 [10.2210/pdb7ZC6/pdb] (Rnf complex). Source data are provided with this paper.

## Source data

Source Data
