# Supplementary material for: Purification and structural characterization of the Na+-translocating ferredoxin: NAD+ reductase (Rnf) complex of Clostridium tetanomorphum
Source: Nat Commun. 2022 Oct 23;13:6315. doi: 10.1038/s41467-022-34007-z (PMC9588780; doi:10.1038/s41467-022-34007-z)

Supplementary data

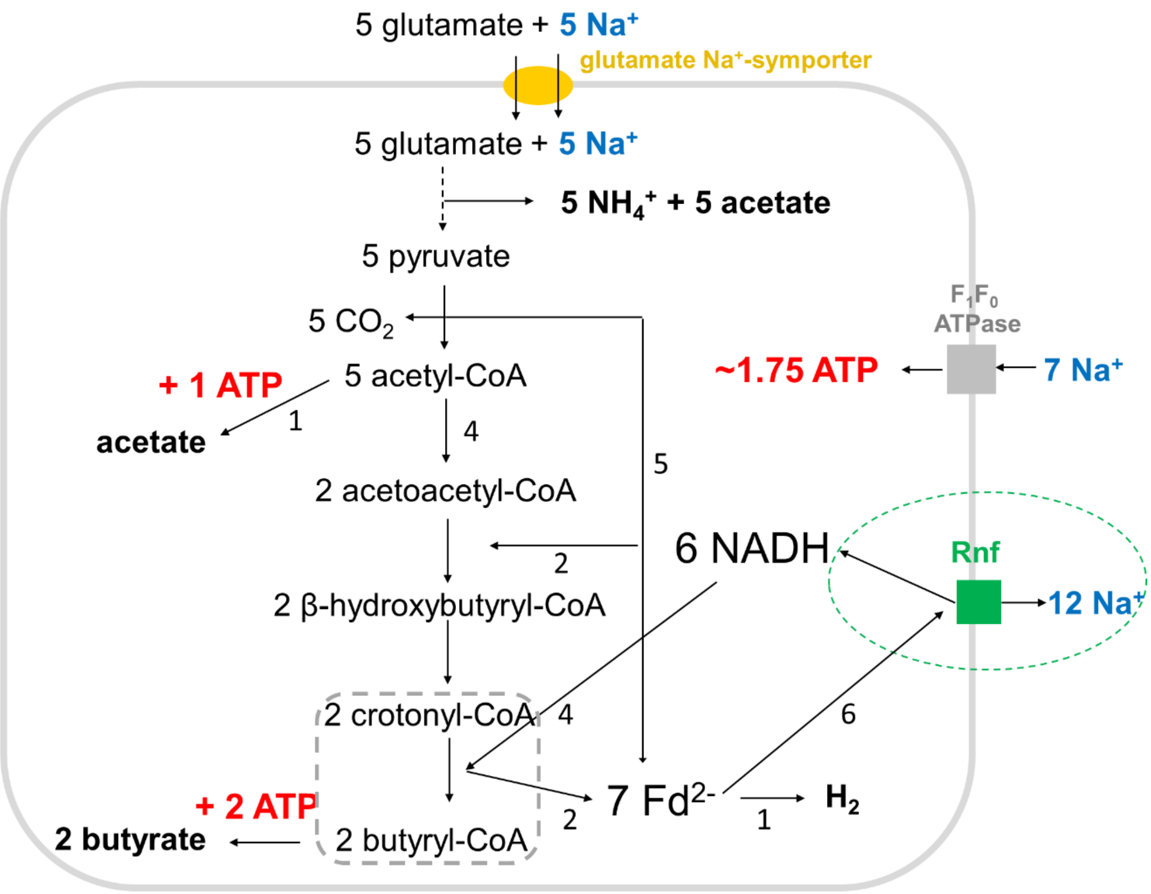

Supplementary Fig. 1: Energy metabolism of the Gram-positive fermenting bacteria *C. tetanomorphum* growing on glutamate. The reaction: 5 glutamate + 6 H_2O + 2 H^+ ⟶ 5 NH_4^+ + 5 CO_2 + H_2 + 6 acetate + 2 butyrate yields a free energy of $\Delta G°'$ = -314 kJ/mol H_2 , which corresponds to ca. 0.95 ATP /mol glutamate[1,2]. Rnf of *C. tetanomorphum* is the only electrogenic enzyme in the cell and produced in large amounts. The generated Na^+ gradient across the cell membrane provides half of the energy for driving the endergonic glutamate transport and ATP synthesis. Energy-rich Fd_{red} for the Rnf reaction is generated by oxidizing pyruvate to acetyl-CoA and by the endergonic oxidation of NADH powered by the exergonic reduction of crotonyl-CoA within the framework of an electron bifurcating process [1,3]. The latter reaction (box surrounded by a gray dashed line) was used for a coupled enzymatic assay to regenerate reduced Fd (Fd^{2-}) for performing the reduction of NAD^+ with Fd^{2-} by Rnf. The assay conditions are given in the Method section. The smooth growth of *C. tetanomorphum* in large amounts and the relative stability and homogeneity of its Rnf, worked out previously [4], makes this enzyme to an attractive candidate for structural and functional studies.

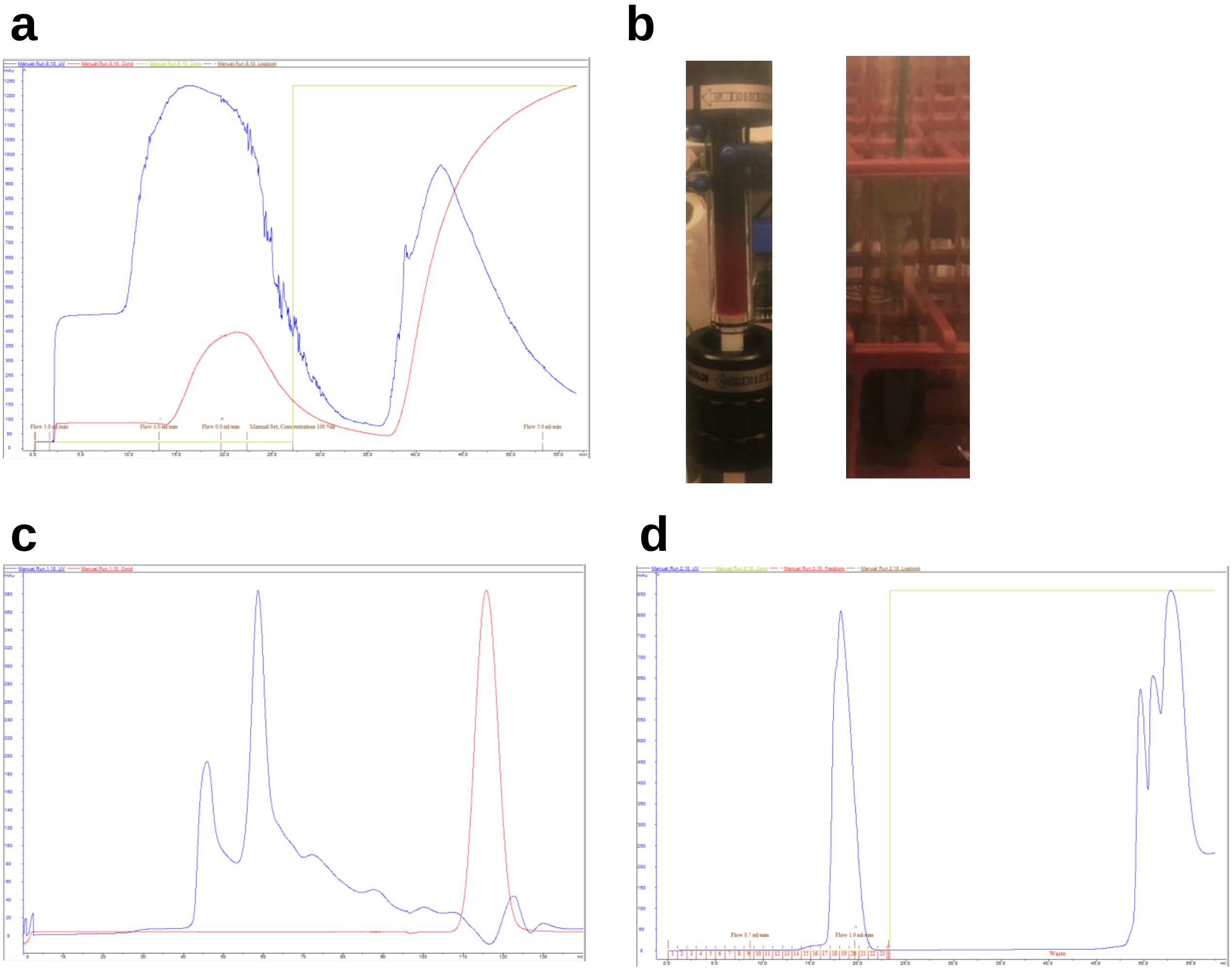

Supplementary Fig. 2: Elution profile of the chromatographic steps. **a** Hydroxyapatite. The solubilized membrane fraction was eluted with the flow-through (red-colored peak). **b** Reactive Red active agarose affinity: Rnf solution was applied to the column with a pipette and washed manually with a NaCl gradient. Rnf was eluted at 2M NaCl. The protein was collected according to the dark brown color of the Fe/S cofactors. **c** Superdex-200 gel filtration. Rnf activity was eluted with 57-62 ml (symmetric red-colored peak). **d** POROS HQ anion exchange resin. The pure Rnf complex is eluted after washing the anion exchange column with buffer A after 18 ml.

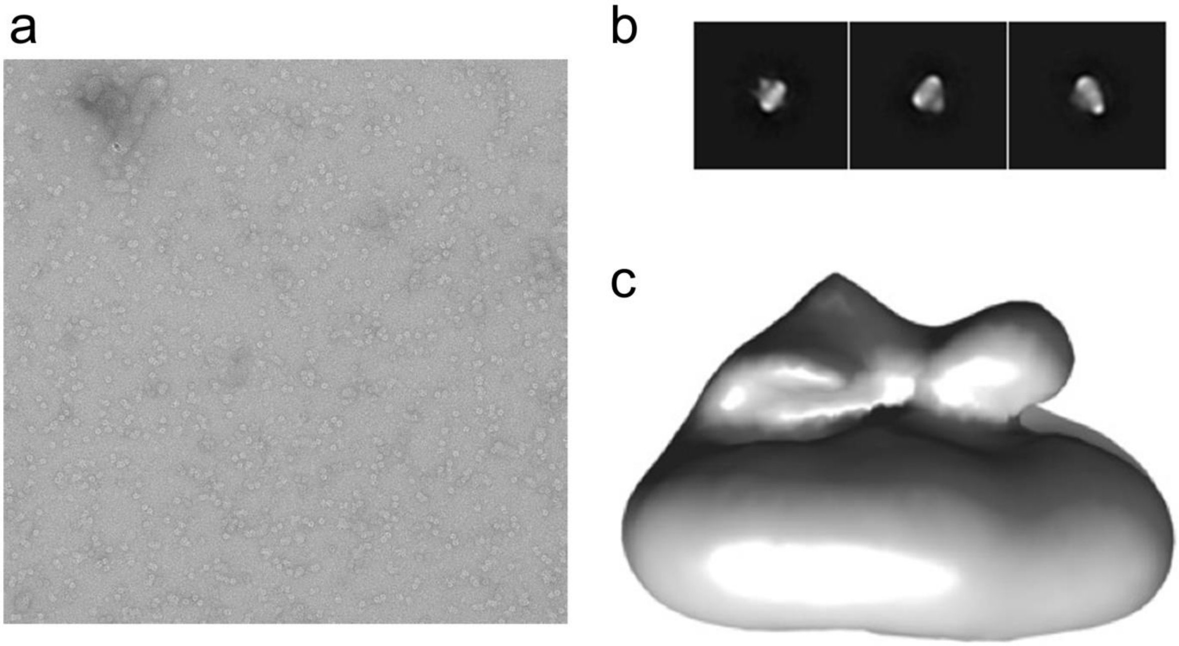

Supplementary Fig. 3: Negative-stain EM analysis of Rnf after purification. a EM micrograph. Data collections were performed eight times. **b** 2D classification. **c** 3D model. For negative-strain electron microscopy grid preparation was done under exclusion of oxygen in an anaerobic tent. The Rnf complex was negatively stained with 2% (w/v) uranyl formate. A 3 µl protein sample (0.02 mg/ml) was pipetted onto freshly glow-discharged carbon-coated copper grids. Electron micrographs were collected using a TecnaiTM Spirit 12G TEM (ThermoFisher – formerly FEI) operating at 120 kV at a nominal 42,000-fold magnification and a calibrated pixel size of 2.68 Å (nominal defocus – 1.50 mm). A set of 200 micrographs were collected on a GATAN 4096 _ 4096 CCD detector by automated data collection with the Leginon software package[5]. Image processing, automatic particle picking, 2D classification and ab initio 3D map generation were performed with cisTEM [6]. The membrane part could be clearly distinguished from the soluble part, potentially consisting of RnfB and RnfC.

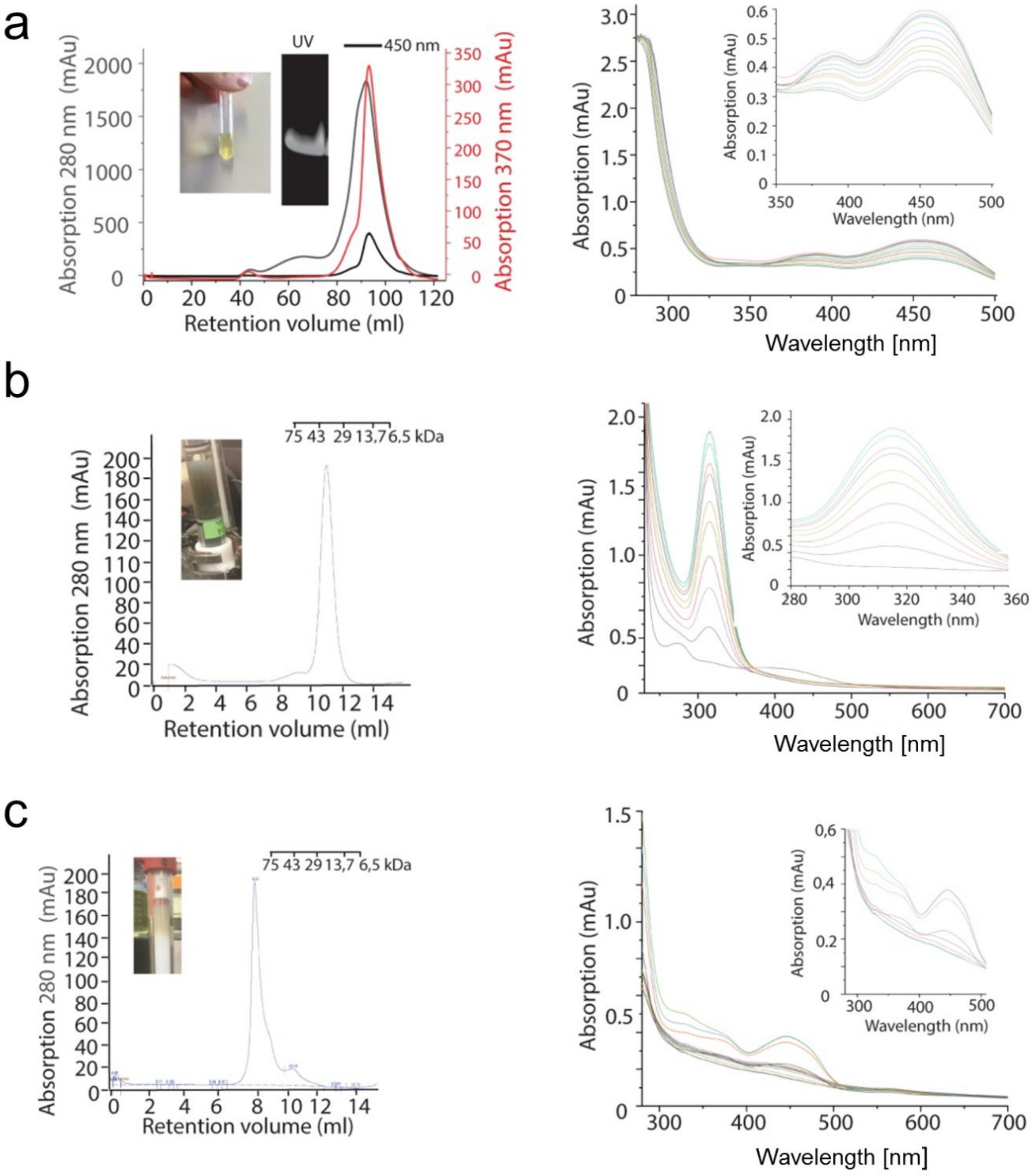

Supplementary Fig. 4: Separated production of the soluble subunits. a RnfG. RnfG of *C. tetanomorphum* was overproduced by co-expression with the *apbE* gene responsible for forming the covalent linkage of FMN with the protein [7,8]. Without co-expression of *apbE* and supplementing the cultivation media with Mg^{2+} and FMN, respectively, RnfG is only produced in an aggregated form devoid of FMN. The *E. coli* cells were opened with a French pressure cell. After cell debris and membrane removal, the protein solution was applied to Ni-NTA agarose in 50 mM Tris-HCl, pH 7.5, 150 mM NaCl and eluted with 250 mM imidazole. Then, the heterologous produced protein was applied to a Superdex-75 column (Hiload® 16/600 Superdex 75® pg, Sigma-Aldrich, Missouri/USA) for further purification and desalting. The yield was 30 mg RnfG from 5 g cells. The protein was pure and homogenous as derived from the gel filtration profile (left panel). Elution was monitored at 280 nm, 370 nm (red) and 450 nm

(small black peak) already indicating the presence of FMN. Its covalent binding became visible in the fluorescent SDS gel (left panel inset). A source data file is provided. The bound FMN could be stepwise reduced by dithionite monitored by UV/Vis spectral changes (right panel). The grey curve is related to RnfG as isolated. 10 x 3 µL of dithionite (20 mM stock solution) was supplemented to the protein resulting in 10 curves (from green ascending to red). **b** RnfB. The soluble part of RnfB from *C. tetanomorphum* was successfully overproduced in *E. coli* BL21 (DE3) containing special plasmids for iron-sulfur cluster formation (pRKISC vectors) [9,10]. Furthermore, *in vivo* iron-sulfur cluster reconstitution with various iron and sulfur sources were carried out as described [11]. RnfB was kept anaerobically during the entire preparation performed by the procedure described for RnfG. RnfB detected at 280 nm is homogeneous (left panel) and pure. The yield was 80 mg RnfB from 5 g cells. The UV/Vis spectra indicate multiple iron-sulfur clusters that were stepwise reducible by dithionite (right panel). The brown curve describes RnfB as isolated. The curve after oxidizing RnfB with 12 µM hexacyanoferrate (III) is drawn in grey and those after adding 8 x 3 µL of dithionite (20 mM stock solution) ranges from violet to green. **c** RnfC. RnfC was produced and purified in the same way as RnfB. The yield was 10 mg RnfC from 5 g cells. All buffers for RnfC purification were supplemented with a flavin mixture (20 pM of riboflavin, FMN and FAD) to avoid loss during purification. RnfC was dominantly present as dimer in the gel filtration profile (left panel). The UV/Vis spectrum (right panel) indicates iron-sulfur clusters and a flavin shown in brown for isolated RnfC. The red and blue curve were obtained after oxidizing RnfC with 6 and 12 µM hexacyanoferrate(III). Addition of 4 x 3 µL of dithionite (20 mM stock solution) results in brown, violet, orange and blue curves. For measuring RnfC activity, NADH was oxidized with hexacyanoferrate (III).

a

```
AKB90213.1   Y. pestis         ---MTESYLTIFIRAVFVENMALNFFLGMCTFLAISKKVETAFGLGLTVTALLAIATPLN    57
TWU82119.1   H. influenca      ----MEHYISLFVKAVFIENMALSFFLGMCTFLAVSKKVSTAFGLGIAVTFVLGIAVPVN    56
ATD25238.1   V. cholerae       ----MEHYISLLVKSIFIENMALSFFLGMCTFLAVSKKVKTSFGLGIAVIVVLTISVPVN    56
GAD71947.1 V. alginolyticus    ----MEHYISLLVKSIFIENMALSFFLGMCTFLAVSKKVKTSFGLGVAVVVVLTIAVPVN    56
sp|Q8TSY0.1 M. acetivorans     MVKMAESLFTIFLEGVFIKNFLLIQFLGLCSFVGVTKDLKSASGMSGAVVFVMAMAATVS    60
ACR23746.1   A woodii          -----MTLIFIMISAIFVNNFVLSRFLGICPFLGVSKQVETAVGMGVAVTFVMALASAIT    55
SQB91611.1 C.tetanomorphum     -----MSIFTIFISALLVNNFVLSRFLGICPFLGVSKKVETATGMGAAVTFVMALAAIMT    55
AKE29923.1   T. maritima       -----MKVFLLFFSAIFVNNFVLARFLGICPFLGVSKRLETATGMGIAVTFVMTVSAAIS    55
CAD44486.1   P. stutzeri       -----MEYALFLIGTVLVNNVVLVYFLGLCPFMGVSGKLDPSLGMGLATTLVMTLGGVSS    55
CAD6013402.1 E. coli           ----MTDYLLLFVGTVLVNNFVLVKFLGLCPFMGVSKKLETAMGMGLATTFVMTLASICA    56
APQ65023.1   S. enterica       ----MTDYLLLFVGTVLVNNFVLVKFLGLCPFMGVSKKLETAMGMGLATTFVMTLASICA    56
AZP58876.1   P. aeruginosa     ----MTELALILVSAILVNNFVLVQFLGLCPFMGVSRKIETAIGLSLATTFVLTLAAMCS    56
                                         ::.  ::::*. *  ***:* *:.::  :. : *:. :.  :: :.

AKB90213.1                     NLIYTYILRENALIDGVDLSFLDFITFIGVLAALVQILEMVLERFLPSLHHTLGAFLP--    115
TWU82119.1                     QLIYANVLKENALIEGVDLSFLNFITFIGVIAGLVQILEMVLDKFMPSLYNALGIFLP--    114
ATD25238.1                     NLVYNLVLKPDALVEGVDLSFLNFITFIGVIAALVQILEMILDRFFPPLYNALGIFLP--    114
GAD71947.1                     NLVYNLVLRENALVEGVDLSFLNFITFIGVIAALVQILEMVLDRFFPPLYNALGIFLP--    114
sp|Q8TSY0.1                    FALYNFILVP------LKLEFLRTIAFIVVIAALVQLVEFIVRKHVPALYRSLGIYLP--    112
ACR23746.1                     YVVQYAILDP------LSLGYLQTIAFILIIAALVQLVEMIIKKSSPSLYQALGVYLPFL    109
SQB91611.1                     FLVERFILIP------LNIQYLSTLAFILVIASLVQFVEMVIKKVSPDLYKALGIYLP--    107
AKE29923.1                     WFLDKLL-IS------TGLEFLRTIVFILVIASFVQFVELFLKKTSPDLYEALGIFLP--    106
CAD44486.1                     WLLERYVLQP------LGIGFLRILSYILVIAGLVQLIEMIIRRVSPPLYRSLGIYLP--    107
CAD6013402.1                   WLIDTWILIP------LNLIYLRTLAFILVIAVVVQFTEMVVRKTSPVLYRLLGIFLP--    108
APQ65023.1                     WLIDTWILIP------LDLIYLRTLAFILVIAVVVQFTEMVVRKTSPALYRLLGIFLP--    108
AZP58876.1                     HILQRYVLRP------LDLEYLRTIGFILVIAVVVQFTEMLVKKTSPLLYRVLGIFLP--    108
                                :   :           : :*  : :* ::*  **: *:.: :  * *:. ** :**

AKB90213.1                     -LLTIHCAIFGATIFMVQRE-YTFTESFVYGTGCGLGWMLAIISMAGLREKMKYS-NIPK    172
TWU82119.1                     -LIAVNCAIFGGVSFMVQRD-YNFPESIVYGFGSGLGWMLAIVALAGLTEKMKYA-DIPA    171
ATD25238.1                     -LITVNCAIFGGVSFMVQRD-YSFAESVVYGFGSGVGWMLAIVALAGIREKMKYS-DVPP    171
GAD71947.1                     -LITVNCAIFGGVSFMVQRD-YNFAESIVYGFGSGVGWMLAIVALAGIREKMKYS-DVPP    171
sp|Q8TSY0.1                    -LITTNCAVLGAVLLNVMND-YDLAQSVVFGVAAGLGYTVAMLMMAAIRERSDLVEVPKS    170
ACR23746.1                     PLITTNCAVLGVALINIQNE-YNFIETIFNGVGAALGFTLAIVLFAGIRERLETS-AVPK    167
SQB91611.1                     -LITTNCAVLGMAVINSNEK-YNLIQSIINSVGAALGFTLALVLLAGIREKMETNEYIPE    165
AKE29923.1                     -LITTNCAILGMVLLNSLMK-LNFVEAVFHALGSGLGFALALVIFAGIREKMDLY-DLPE    163
CAD44486.1                     -LITTNCAVLGVPLISVREG-HRLAEAGLFGLGSALGFTLVMVIFAGLRERLALA-SVPA    164
CAD6013402.1                   -LITTNCAVLGVALLNINLG-HNFLQSALYGFSAAVGFSLVMVLFAAIRERLAVA-DVPA    165
APQ65023.1                     -LITTNCAVLGVALLNINLG-HHFLQSALYGFSAAVGFSLVMVLFAAIRERLAVA-DVPA    165
AZP58876.1                     -LITTNCIVLGVALLNANKAEYGFLQATTQGFGAGLGFSLVLVLFAALRERIAIA-DVPA    166
                                *:: :* ::*   :        :  :   . ...:*: :.:: :*.: *:

AKB90213.1                     GLQGLGIVFMTAGLMSLGFMSFSGIRL--       199
TWU82119.1                     GLKGLGITFISVGLMALGFMSFSGIQL--       198
ATD25238.1                     GLRGLGITFITAGLMALGFMSFSGVQL--       198
GAD71947.1                     GLRGLGITFITVGLMALGFMSFSGVQL--       198
sp|Q8TSY0.1                    VGRGVTYAFFIATIMSMSFVNFFGVIPLE       199
ACR23746.1                     ALEGFPIALLTAGLMAIAFLGFSGMKLG-       195
SQB91611.1                     ALKGLPITLVTAGLMAIAFLGFQGLI---       191
AKE29923.1                     PFKGTAIALITAGLLSLAFMGFQGMVKL-       191
CAD44486.1                     AFAGAPIAFVTAGLLAMAFMGFAGLI---       190
CAD6013402.1                   PFRGNAIALITAGLMSLAFMGFSGLVKL-       193
APQ65023.1                     PFRGNAIALITAGLMSLAFMGFSGLVKL-       193
AZP58876.1                     PFRGAAIGMITAGLMSLAFMGFSGLVRP-       194
                                  *    :. . ::::.*:.* *:
```

b

```
EGT68456.1 E. coli             -MNAIWIAVAAVSLLGLAFGAILGYASRRFAVEDDPVVEKIDEILPQSQCGQCGYPGCRP    59
APQ65024.1 S  enterica         -MNTIWIAVGALALLGLVFGAILGYASRRFAVEDDPVVEKIDAILPQSQCGQCGYPGCRP    59
SUD83625.1 P. stutzeri         -MSLVLIAILALLALCLVCGAILGFAAVRFRVEGDPIAEQINALLPQTQCGQCGYPGCKP    59
AZP58875.1 P. aeruginosa       -MNGVFLAIGALLPICLAGGALLGYAAVRFRVQGDPVAEQVNALLPQTQCGQCGYPGCKP    59
AGL49173.1 T. maritima         --MEVIYSTLLLAVLGFGFGAFLAYSAQRFKVEEDPRVKMITEVLPGINCGACGFAGCEA    58
sp|Q8TSX9.1 M.acetivorans      MSSVLINSIAVLAGLGFAVGVMLVIASKVFKIDSNPLIDDVASLLPGANCGGCGFAGCAA    60
ACR23747.1 A. woodii          MLNAILVPVGILGVFGLIFGIGLAIAAKVFEVYEDPRVPLVRAALPGANCGGCGLPGCDA    60
SQB91612.1 C.tetanomorphum    -MEGLLFPVLSLGGLGVVFGLLLGYASKKFAVEVDERVPMVRAALPGANCGGCGFAGCDA    59
                                   :      :  : .  *  *  ::  * :  :     :   **  :** **  **
```

```
EGT68456.1                   YAEAISCNGEKINRCAPGGEAV-MLKIAELLNVEPQPLDGE---------AQELTPARMV    109
APQ65024.1                   YAEAVGLQGEKINRCAPGGEAV-MLKIAELLNVEPQPCDGE---------EQQAAPVRML    109
SUD83625.1                   YAEAIA-GGDKINKCPPGGEAT-IQALADLLDVEPEPLDAA---------EGEVPP--RV    106
AZP58875.1                   YAEAIA-AGDKINKCPPGGEAT-IRALADLLDLEPEPLDAA---------E-ETPP--RV    105
AGL49173.1                   YAKAIVKGQAETNRCLPGRPQGVEEKIKKILEEYKNVSS---------------------    97
sp|Q8TSX9.1                  CAEAIVEQGAPVNSCPVGGFEV-AKQIGALLGQEVTESEKEFPFVRCQGGNQHCTT--LY    117
ACR23747.1                   LAANIVGGSAAIDACPVGGASC-AAAVAEIMGMEAGSAVKKVATVICQGTCETAPN--RA    117
SQB91612.1                   YADAVVNAGAKPNGCPVGGAAC-AAKIAEIMGVVVDSSEPKKAYVKCQGTCDKAKE--KY    116
                              *  :        : *   *          :   ::

EGT68456.1                   AVI------------------DENNCIGCTKCIQACPVDAIVGATRAMHTVMSDLCTGCN    151
APQ65024.1                   AVI------------------DENNCIGCTKCIQACPVDAIVGATRAMHTVMSDLCTGCN    151
SUD83625.1                   AYI------------------REAECIGCTKCIQACPVDAIVGAAKQMHTVIVTECTGCD    148
AZP58875.1                   AYI------------------REAECIGCTKCIQACPVDAIVGAARLMHTVIADECTGCD    147
AGL49173.1                   ------------------------------------------------------------    97
sp|Q8TSX9.1                  DYHGVENCKVAL-MLCDSRKGCTYGCLGLGTCVQACQFGALSMGEDGFPVVNKALCTSCG    176
ACR23747.1                   EYYGEMDCREAM-IASGGSKGCRYGCLGYGTCKAVCPFDAIVIGEDGLPKVDPEKCTSCG    176
SQB91612.1                   EYYGAMTCVDAANIPGAGSKTCGFGCLGLGSCVQVCAFDAIHV-ENGIAVVDEEACTGCG    175

EGT68456.1                   LCVDPCPTHCISLQPVAETPDSWKWDLNTI-PVR--------------------IIPVEH    190
APQ65024.1                   LCVDPCPTHCIELRPVNETPDSWKWDLNTI-PVR--------------------IIPVEQ    190
SUD83625.1                   LCVEPCPVDCIDMIELGSNLQSWKWDQPLA-PGQ--------------------LIASDR    187
AZP58875.1                   LCLEPCPVDCIEMRETPDDVRHWKWPQPSP---R--------------------LIASDR    184
AGL49173.1                   ------------------------------------------------------------    97
sp|Q8TSX9.1                  NCIAACPNGVLTFARDSEKVHVLCRSHDKGKDVKAVCEVGCIGCKKCEKECPAGAIRVTE    236
ACR23747.1                   KCVEACPKSIMTLVPEAQEVIVKCHNFDKGKIARLSCTTACIACGACVKACRFDAITVEN    236
SQB91612.1                   ACVSICPKSVIELTPMSKKVRISCNSHDKGIEVKNACSVGCLSCGLCVRNCPSEAITMVN    235

EGT68456.1                   HA----------------------------------------------------------    192
APQ65024.1                   HA----------------------------------------------------------    192
SUD83625.1                   EQAA--------------------------------------------------------    191
AZP58875.1                   ERAA--------------------------------------------------------    188
AGL49173.1                   ------------------------------------------------------------    97
sp|Q8TSX9.1                  FLAEIDQEKCTACGACVAICPQKAIELR--------------------------------    264
ACR23747.1                   NCAKIDYDKCRQCYECVDKCPMNCISGDVEYGKSTAYIIEENCIACGLCAKNCPVNAITG    296
SQB91612.1                   NLPVIDYDKCTQCGVCVGKCPTKAIVNLNTNVSKEA---SNN------------------    274

EGT68456.1                   -------------------------------------  192
APQ65024.1                   -------------------------------------  192
SUD83625.1                   -------------------------------------  191
AZP58875.1                   -------------------------------------  188
AGL49173.1                   -------------------------------------  97
sp|Q8TSX9.1                  -------------------------------------  264
ACR23747.1                   EIKKPPYVIDHDMCIGCGICFDKCRKSAIEMRPNKTK  333
SQB91612.1                   -------------------------------------  274
```

C

```
WP_000103965.1 V. cholerae  MSTIIFGVVMFTLIILALVLVILFAKSKLVPTG------DITISINGDPEKAIV------     48
AAM04101.1 M. acetivorans  ------------------------MKRSLHSKEVANLSDV--IKIDKLPEKAIIPMRQHD     34
AKE29919.1 T. maritima     ---------------------MLTFKGGVHPPELKEWSKDKPIERAPLPQKVFVFLSNHA     39
ACR23742.1 A. woodii       -----------------MNVKHGTFKGGIHPPYRKESTAEVPLGFGKKPEMVIIPMSLHI     43
KAJ52772.1 C. tetanomorphum -------------------MELLTFKNGVHPPHGKHYTENKPIEEYLPKGDIVIPMSQHI     41
CAD6007421.1  E. coli      ---------MLKLFSAFRKNKIWDFNGGIHPPEMKTQSNGTPLRQVPLAQRFVIPLKQHI     51
ALE62000.1 S. enterica     ---------MLKLFSAFRKDKIWVFDGGIHPPEMKTQSNGTPLRQVPLAPRFVIPLKQHI     51
AEJ04398.1 P. stutzeri     ----------------MTSLKVWDIHGGIHPPEHKELSNRTPIQPAPLPKRLILPLAQHL     44
KYO98541.1 P. aeruginosa   ------------------MSALHAFPGGLCLPANKERSTALPIQQAPLAQRYIVPLGQHI     42
                                                 :             :         .:

WP_000103965.1              ------------------------------------------------------------     48
AAM04101.1                  GIACAPLVKKGAEVIVGQKLGECEGSDLAYVHSPFCGTVNSIELMP--NPS-GKRILSVV     91
AKE29919.1                  GNPAKPVVSPGDEVKTGQVIGEPEGFISAYLHSPVTGRVLEIKKIL--HPILGKPIEAIV     97
ACR23742.1                  GAPCTPIVKKGDTVFLGQRVGEPNGFVSVPVHASVSGKVIAVEERP--HAS-GDRVMSVV     100
KAJ52772.1                  GAPAEPIVKKGDRVLVGQKIGEAKGFVSANIHASVSGTVKNVAPVT--LFN-GVKSTAVI     98
CAD6007421.1                GAEGELCVSVGDKVLRGQPLTRGRGKM-LPVHAPTSGTVTAIAPHSTAHPS-ALAELSVI     109
ALE62000.1                  GAEGELCVSVGDRVLRGQALTRGRGRM-LPVHAPTSGTVIAIAPHSTAHPS-ALAELSVI     109
AEJ04398.1                  GAPAEPCVALGEHVLKGQQIAVASGFVSAALHAPTSGVVSFIGPQPYPHVS-GMLANAIV     103
KYO98541.1                  GAPARPCVEVGQAVLKGQTIALPDGTVSAALHAPTSGTVVAIGAHPYPHAS-GLPAPAIV     101
```

```
WP_000103965.1        ---------------------TQPGGKLLTALAGAGVFVSSACGGGGSCGQCRVKIKSGG   87
AAM04101.1            LTPSECAQTVD-----FVPEKDAPPSRLIEIIKEAGI-----VEY----YEKPTYLALKP   137
AKE29919.1            IERTSDDEWVHI---ETGDFERMSKEEILEIIKKAGI-----VGLGGAMFPTHVKLSPPP   149
ACR23742.1            IESDGL-DTIDPSIKPYGTLEDMDADAIKKMVLNAGI-----VGLGGATFPTHVKLAIPP   154
KAJ52772.1            IENDGQYEEIETEKRDYTK---LSNEEIINIIKEAGI-----VGMGGATFPTHVKLAPPP   150
CAD6007421.1          IDADGEDCWIPRD--GWADYRTRSREELIERIHQFGV-----AGLGGAGFPTGVKLQGG-   161
ALE62000.1            IDADGEDRWIERE--GWSDYRAHSREALIERIHQYGV-----AGLGGAGFPTGVKLQGG-   161
AEJ04398.1            IDSDGEDRWIELE--PHLDYRALERPALLELIRQAGI-----SGLGGAGFPTAVKLTPPP   156
KYO98541.1            IASDGLERWTELH--PCPDFRAESPLALLERIRAAGI-----GGLGGAGFPTAAKLAARP   154
                                         :   :   *:                .  :

WP_000103965.1        GDILPTELDHISKGEA--REGERLACQVAVKADMDLELPEEIFGVKKWECTVISNDNKAT   145
AAM04101.1            GKRIDTLLMNATF--PLIT---HAYLSSLDKVLEGFKLMLEASGISR--GVIVLRADDKE   190
AKE29919.1            EKKVDTLIVNGAECEPVLTIDHRLMLERAEDILQGILIMMKVLGVQK--AVVGVESNKMD   207
ACR23742.1            DKKVDCVVLNGAECEPYLTADHHLMTSQAEKVVMGLKLAMKSVGVEK--GFIGVEDNKTD   212
KAJ52772.1            DKNIDSIVVNAAECEPYLTCDHRMMLEKTNEIVEGLKIVLKLFPKAT--GYIGIEDNKMN   208
CAD6007421.1          GDKIETLIINAAECEPYITADDRLMQDCAAQVVEGIRILAHILQPRE--ILIGIEDNKPQ   219
ALE62000.1            GDKITTLIINAAECEPYITADDRLMQDCAAQIVEGIRILAHILQPRE--VLIGIEDNKPQ   219
AEJ04398.1            TQRIRTLIINGTECEPYITADDLLMREKAAELVAGIEILAHLIQPQD--VLIGIEDNKPE   214
KYO98541.1            AEKIHTLVVNGAECEPYISADDLLMRERATQVLGGIDILVQILRPEE--VLVGIEDDKPE   212
                       . :   : : :              .   .   .: :  .          :  . :.

WP_000103965.1        FIKELKLAIPDGGSVPFRAGGYIQIEAPAHHVKYADFDVPEKYRGDWDKFNLFRYESKVD   205
AAM04101.1            SIKAFKNAKVDGKPLTVAPI--VGM----RHADYYLEDVEDQII--VVAAGKITYTPTMM   242
AKE29919.1            AYHNLKKVFKG-YPVDVALL-------------------------------RTKYPQGAE   235
ACR23742.1            AIEALVKAIGNDSRLEVYSL-------------------------------HTKYPQGAE   241
KAJ52772.1            AIKAMQEAVKNIANIEVKAV-------------------------------KTKYPQGAE   237
CAD6007421.1          AISMLRAVLADSNDISLRVI-------------------------------PTKYPSGGA   248
ALE62000.1            AISMLRAVLADAHDISLRVI-------------------------------PTKYPSGGA   248
AEJ04398.1            AIAAVRAAIGER-PFVLKVF-------------------------------PTKYPSGGE   242
KYO98541.1            AIAALGAALGER-PYRIVAL-------------------------------PTRYPSGGE   240
                          .  .        .                                      *

WP_000103965.1        EPII-----RAYSMANYPEEFGIIMLNVRIATPPPNNPNVPPGQMSSYIWSLKAGD----   256
AAM04101.1            NLLSANVMGRKLPLGYEPPDVHVVVCGVKSAKAVYDAINEGKPYLESAV-TV-TG-AVNN   299
AKE29919.1            KQLIYAITGRMVPRGGLPMDVGVVVQNVGTCVAVKEAVVDGKPLVERGM-TV-SGDAVKN   293
ACR23742.1            KQLIAAITGREVPSGALPADAGVVVMNVGTAAQIAESMITGLPLYKRYL-TC-TGDAIKN   299
KAJ52772.1            KQLIYAITKREVPSGGLPADAGCIVQNVDTIYEIYNAVVNGKPLTSRVV-TV-TGDAIKE   295
CAD6007421.1          KQLTYILTGKQVPHGGRSSDIGVLMQNVGTAYAVKRAVIDGEPITERVV-TL-TGEAIAR   306
ALE62000.1            KQLTRILTGKQVPHGGRSSDIGVLMQNVGTAYAVKRAVIDGEPITERVV-TL-TGEAVSR   306
AEJ04398.1            KQLIQILTGEEVPSGGLPADIGMLCQNVGTCVAIHDAVLLGKPLISRIT-TL-TGEALAR   300
KYO98541.1            RQLIQLLTGREVPADGLPADIGILCQNVGTLAAVHDAVVLGRPLISRIT-TL-AGGALER   298
                      . :      .         :   :  .*                 .    :  :*

WP_000103965.1        ----KCTISGPFGEFFAK----DTDAEMVFIGGGAGMAPMRSHIFDQLKRLKSKRKMSYW   308
AAM04101.1            PKTVIVKFGTPIKDVIEACGGYKGEPGKVIVNGSMGGVAV------------------YT   341
AKE29919.1            QKNLIVRIGTPVKDVIDYCGGIDENTERVILGGPMMGISI------------------TN   335
ACR23742.1            PQTIEIRIGVPFQSVIDQCGGFSSEPGKVISGGPMMGVTQ------------------FV   341
KAJ52772.1            PKNLRFKIGTSVRELVEAAGGFAEEPLKVISGGPMMGMAM------------------YS   337
CAD6007421.1          PGNVWARLGTPVRHLLNDAGFCPSADQMVIMGGPLMGFTL------------------PW   348
ALE62000.1            PGNVWARLGTPVRHLLNDAGFCPSADQMVIMGGPLMGFTL------------------PW   348
AEJ04398.1            PMNVEALIGTPAGELLAFAGLEQNRLNRLIMGGPMMGFTL------------------PS   342
KYO98541.1            PMNVEVLIGTPVHELLAFAGLAEGRLERVLMGGPMMGFAL------------------PD   340
                             :.     ..            :: .*

WP_000103965.1        YGARSKREMFYVEDFDGLAA---------ENDNFVWHCALS------DPQPEDNWTGYTG   353
AAM04101.1            DEAPV------VKNTVGIVVQTEAEVL-RDEATVCIHCARCVDVCPMNLLPGRIAAMADM   394
AKE29919.1            LDIPV------MKGTSGITAFLP-KKS--RPQKPCIRCSECVQVCPMNLQPYLLYLLSTK   386
ACR23742.1            TDIPV------MKGTSGILCLTK-ESAKIATPSNCIHCGKCVGVCPIHLQPLNIAEYSQR   394
KAJ52772.1            LDVPS------TKGTSGVLCLTK-KVAEIEEESNCINCGKCVQVCPMNLMPTKLATASAV   390
CAD6007421.1          LDVPV------VKITNCLLAPSANELGEPQEEQSCIRCSACADACPADLLPQQLYWFSKG   402
ALE62000.1            LDVPV------VKITNCLLAPSVTEMGAPQEEKSCIRCSACADACPADLLPQQLYWFSKG   402
AEJ04398.1            LDVPV------IKTTNCLLASTLEELPPPPALPCIRCGECAEACPVSLLPQQLHFFALG   396
KYO98541.1            LSVPL------IKTCNCLLAGDATELPEPVPAMPCIRCGDCAQVCPVSLLPQQLHFFALG   394
                                  :    :                  .*. .         *

WP_000103965.1        FIHNVLYENYLKDHEAPEDCEYYMCGPPMMNAAVINMLKNLGVEEENILLDDFGG-----   408
AAM04101.1            GMFDRCREYFALNCIECGECA-VVCPAKR------HLVQLIRYSKLQIMNQKNETVEATE   447
AKE29919.1            RKYDEAVENGLMDCIECGSCT-YTCPSKI------EHVRYIKLAKTVYRATRRGRR----   435
ACR23742.1            NMWDKCESNNAMDCIECGSCS-YICPAKR------TLVSSIRVAKREIIAQRRKGN----   443
KAJ52772.1            SNLDMFNEFSGRDCIECGCCS-FVCPARR------HLLQRIRSGKKAVSKKK--------   435
CAD6007421.1          QQHDKATTHNIADCIECGACA-WVCPSNI------PLVQYFRQEKAEIAAIRQEEKRAAE   455
ALE62000.1            QQHDKATAHHIADCIECGACA-WVCPSNI------PLVQYFRQEKAEINAIRLEEKRAAE   455
AEJ04398.1            QEHEQLKAHHLFDCIECGACA-YVCPSSI------PLVQYYRAAKAEIRELEQKQQKAEH   449
KYO98541.1            DEHEQLLAHNLFDCIECGACA-YVCPSSI------PLVQYYRASKAEIREQRQKLLKAEQ   447
                         :        :      *    *            :      :
```

```
WP_000103965.1    ------------------------------------------------------------    408
AAM04101.1        ------------------------------------------------------------    447
AKE29919.1        ------------------------------------------------------------    435
ACR23742.1        ------------------------------------------------------------    443
KAJ52772.1        ------------------------------------------------------------    435
CAD6007421.1      AKARFEARQARLEREKAARLERHKSAAV----QPAAKDKDAIAAALARVKEKQAQATQPI    511
ALE62000.1        AKARFEARQARLEREKAARLARHKSAAV----QPAAKDQDAIAAALARVKEKQAQATQPV    511
AEJ04398.1        SKQRFELRQERLRRAEEQKEAERKARAERAARAKAAQGESAQVVS--------DTTAA--    499
KYO98541.1        SRERFEQRQARLRRDEERRAAERAQRAEKAALARAAQAEREEAAP--------ATAVDPV    499

WP_000103965.1    ------------------------------------------------------------    408
AAM04101.1        ------------------------------------------------------------    447
AKE29919.1        ------------------------------------------------------------    435
ACR23742.1        ------------------------------------------------------------    443
KAJ52772.1        ------------------------------------------------------------    435
CAD6007421.1      VIKAGERPDNSAIIAAREARKAQARAKQAELQQTNDAATVADPRKTAVEAAIARAKARKL    571
ALE62000.1        VIQAGSQPDNSAVIAAREARKAQARAKQAAHPMADSAIPGDDPSKAAVEAAIARAKARKQ    571
AEJ04398.1        ---------------TPSAAPKS--------------GLSETQKKLKIEASMAQVALKKA    530
KYO98541.1        QA---------AIERARARKQAG--------------SGSERLKRLKIEASMARVALKKA    536

WP_000103965.1    ------------------------------------------------------------    408
AAM04101.1        ------------------------------------------------------------    447
AKE29919.1        ------------------------------------------------------------    435
ACR23742.1        ------------------------------------------------------------    443
KAJ52772.1        ------------------------------------------------------------    435
CAD6007421.1      EQQQANA----------------------------------------EPEQQVDPRKAAV    591
ALE62000.1        EQ-QAGS----------------------------------------EPAEPVDPRKAAV    590
AEJ04398.1        EKQLAAHDTPELQAQVADLRKAAEAAQQALDAVM---QENASAAPTVSPADDEALKKAKI    587
KYO98541.1        EKQLLGHDTPEQHGLVAELRAAAEAADKALADAEASLPRDLPSAPPAALDDEAELKKAKA    596

WP_000103965.1    ------------------------------------------------------------    408
AAM04101.1        ------------------------------------------------------------    447
AKE29919.1        ------------------------------------------------------------    435
ACR23742.1        ------------------------------------------------------------    443
KAJ52772.1        ------------------------------------------------------------    435
CAD6007421.1      EAAIARAKARKLEQQQANAEPEE---QVDPRKAA---VEAAIARAKARKLEQQQANAEPE    645
ALE62000.1        EAAIARAKARKQEQ-QAGSEPVE---AVDPRKAA---VEAAIARAKARKQEQQ-TGSEPA    642
AEJ04398.1        EAAMLKAQIRKLEKL--EAPDDDQQAELARLRQQLHEAEQALAAAQ-------SAAPAPA    638
KYO98541.1        QAAMARAQLKRSEKAFGEAPGAEQRATLDELRAEVERCEATLARLE-------RHAPKPA    649

WP_000103965.1    ------------------------------------------------------------    408
AAM04101.1        ------------------------------------------------------------    447
AKE29919.1        ------------------------------------------------------------    435
ACR23742.1        ------------------------------------------------------------    443
KAJ52772.1        ------------------------------------------------------------    435
CAD6007421.1      QQ-----VDPRKAAVEAAIARAKARKREQQPAN---------------------------    673
ALE62000.1        EA-----VDPRKAAVEAAIARAKARKQEQQT-G---------------------------    669
AEJ04398.1        AKPA-DDEALKKAKIELAMKRAELKKAEKAGAQEAELSRLRDALQAAEQALHAAEDASHK    697
KYO98541.1        APGDDGQAALKRAKIALVGKRAALKKAEQAGVMDSELERLRGELQAAERDLHAAEDACGK    709

WP_000103965.1    ------------------------------------------------------------    408
AAM04101.1        ------------------------------------------------------------    447
AKE29919.1        ------------------------------------------------------------    435
ACR23742.1        ------------------------------------------------------------    443
KAJ52772.1        ------------------------------------------------------------    435
CAD6007421.1      ------AEPEEQVDPRKAA—VEAAIARAKARKLEQQQANAVPEEQVDPRKAAVAAAIAR     725
ALE62000.1        ------SEPAEPIDPRKAA--VEAAIARAKARKQEQQ-AGSEPAEPADPRKAAVAAAIAR    720
AEJ04398.1        PAPELVRTSKPGIDERQRELKTEVAFARADLRKLERDEQT-EPT--------TLEAARLR    748
KYO98541.1        PAPELVRIDKRPVDPRTRELKTELAYARAALKKLERLANA-DAA--------ALAAARAR    760

WP_000103965.1    ------------------    408
AAM04101.1        ------------------    447
AKE29919.1        ------------------    435
ACR23742.1        ------------------    443
KAJ52772.1        ------------------    435
CAD6007421.1      AQAKKAAQQKVVNED---    740
ALE62000.1        VQAKKAAQQQVVNED---    735
AEJ04398.1        LSEA----ERQLADYQQS    762
KYO98541.1        LSAA----ERALTEHGTE    774
```

d

```
GAD71712.1 V. alginolyticus MSLKHYFEKATPKFGPGGKYHKFYPLFEAVETIFYTPGKVNQGMTHVRDSIDLKRIMIMV       60
AAX87224.1 H. influenza     MGLKNLFEKMEPAFLPGGKYSKLYPIFESIYTLLYTPGTVTHKNTHVRDALDSKRMMITV       60
AKB05379.1 V. cholerae       MGLKKFLEDIEHHFEPGGKHEKWFALYEAAATLFYTPGLVTKRSSHVRDSVDLKRIMIMV      60
AKB88576.1 Y. pestis        MGLKNFLEKIEHHFEAGGKLEKYYPLYEAAATIFYTQGKVTPGASHVRDAIDLKRMMILV       60
WP_011020707.1 M.acetivorans-----------------------------------MTSFTVSPPPHIKKKIFIKNLIWSR       25
AKE29920.1 T. maritima      ------------------------------------MKLISAYAPHLREEDDVRKIMLDV       24
AFA48979.1 A. woodii        ---------------------------------MNELNLTVSSSPHIRAKHSTASIMQNV       27
SQB91608.1 C. tetanomorphum --------------------------------MSETTMYTVSSSPHIRAKDTTQSIMRDV       28
CAC03727.1 P. stutzeri      -----------------------------------------------MIRSSVDRIMLHV       13
AZP58873.1 P. aeruginosa    ------------------------------------MALPRPTSPHARGSNRTPAIMRLV       24
EFF12894.1 E. coli         ------------------------------------MVFRIASSPYTHNQRQTSRIMLLV       24
KPF39873.1 S. enterica      ------------------------------------MVFRIASSPYTHNQRQTSRIMLLV       24
                                                                                   ::

GAD71712.1                  WLAVFPAMFWGMYNIGQQASDALLYGMEQASAQQALVSSWQLSWVW-GSLDAVSQAGWES       119
AAX87224.1                  FLALFPAIFYGMYNVGNQAIPALNQLG---NLDQLIANDWHYALASSLGLDLTANATWGS       117
AKB05379.1                  WLAVFPAMFWGMYNAGGQAIAALNHLYSGDQLAAIVAGNWHYWLTEMLGGTMSSDAGWGS       120
AKB88576.1                  WFAVFPAMFWGMYNVGYQAIPALNQLYSGAELQQVIAGDWHYRLAQMLGASLTPDAGWAS       120
WP_011020707.1              IVALLPISAAAVYFFGFAALGNIIAS----ILG---------------------------       54
AKE29920.1                  LIALSPAVIGAAYFFGWYALFLCIAGA---VIG---------------------------       54
AFA48979.1                  IIALLPALAVAGYVFGLWALALVAIC----VIS---------------------------       56
SQB91608.1                  VIALLPATIAGVYFFKLQGLLVILAS----VLS---------------------------       57
CAC03727.1                  CLALLPTTAWGLYLFGWPAIYLWLLT----CAS---------------------------       42
AZP58873.1                  LGACVPGLLTLTWLYGPGTLLNLAWA----SLV---------------------------       53
EFF12894.1                  LLAAVPGIAAQLWFFGWGTLVQILLA----SVS---------------------------       53
KPF39873.1                  LIAALPGIAAQTWFFGWGTLFQIVLA----AIT---------------------------       53
                              *  *      :

GAD71712.1                  KMWLGATYFLPVYATVFIVGGFWEVLFAIVRK-----HEVNEGFFVSSILFALILPPTIP       174
AAX87224.1                  KMALGAIFFLPIYLVVFTVCTIWELLFSVVRG-----HEVNEGMFVSTILFALIVPPTLP       172
AKB05379.1                  KMLLGATYFLPIYATVFIVGGFWEVLFCMVRK-----HEVNEGFFVTSILFALIVPPTLP       175
AKB88576.1                  KMLLGAAYFLPIYAVVFLVGGFWEVVFSIVRK-----HEINEGFFVTSILFSLIVPPTLP       175
WP_011020707.1              -------------------AVGIEFVIQKAFNKKL--TIMDGNAIYLGLLLALICPPTLP       93
AKE29920.1                  -------------------ELFDIFVMRYLRGVKD--FVPDGSGAVTGLLLAMNVSTRLP       93
AFA48979.1                  -------------------SVATEAVIQKLLKKPI--TVNDWSAVVTGVLLAFNLPINAP       95
SQB91608.1                  -------------------CVVAEYIWQKASKKKV--TVGDYSAVVTGLLLAFNVPASIP       96
CAC03727.1                  -------------------AVACEAACLYLLGRPLR-RLLDGSALLSGWLLALTLPPWAP       82
AZP58873.1                  -------------------ALACEAAMLALRKRPPGVFLKDGSALVTALLLAVALPPYAP       94
EFF12894.1                  -------------------ALLAEALVLKLRKQSVAATLKDNSALLTGLLLAVSIPPLAP       94
KPF39873.1                  -------------------ALVAEAIVLRLRKQSVASHLQDYSALLTGLLLAVSIPPLAP       94
                                                                    : .      *::.      *

GAD71712.1                  LWQAALGITFGIVVAKEIFGGTGRNFLNPALAGRAFLYFAYPANMSGGAV----------       224
AAX87224.1                  LWQAALGITFGIIVAKEIFGGVGRNFMNPALAGRAFLFFAYPAQISGDTV----------       222
AKB05379.1                  LWQAALGITFGVVVAKEVFGGTGRNFLNPALAGRAFLFFAYPAQISGDLV----------       225
AKB88576.1                  LWQAALGISFGVVIGKEIFGGTGRNFLNPALAGRAFLFFAYPAQISGDLV----------       225
WP_011020707.1              AWMIFIGGAFAVGVGKHAFGGIGSYTFHPSLAAWVFLSLAWAQDMLPGTIPILSS-----       148
AKE29920.1                  FWAFLLGLVFALGIGKHVFGGLGQNIFNPALVGRAFLLISFPTYMTTWVVPGAGF-----       148
AFA48979.1                  WWIGVVGSVFAIAIVKQCFGGLGQNFINPALAARAFLLASWPGHMTSTAYI---------       146
SQB91608.1                  LWIPVVGGFFAIIVVKQFFGGLGQNIVNPALAARAFLLASWPVQMTSWT-----------       145
CAC03727.1                  WWIAVGGSMFAIGIGKQLYGGVGQNVFNPAMLARVALLIAFPLQMTTWALPLPLGTEGAP       142
AZP58873.1                  WWLTLVATFFALVFGKHLYGGLGQNPFNPAMLGYVVALVSFPLEMTRWPSPDSAL--GLP       152
EFF12894.1                  WWMVVLGTVFAVIIAKQLYGGLGQNPFNPAMIGYVVLLISFPVQMTSWLPPHEIA-VNIP       153
KPF39873.1                  WWMVVLGTGFAIIIAKQLYGGLGQNPFNPAMIGYVVLLISFPVQMTSWLPPYEIA-ATTP       153
                             *    .  *.: . *. :** *   .:*:: . .    ::   :

GAD71712.1                  -----------------------WVAADGYSGATPLSQWYEGGQSALI-NNMTGQTISWG       260
AAX87224.1                  -----------------------WTAADGFSGATALSQWSQGGQGALQ-HTVTGAPITWM       258
AKB05379.1                  -----------------------WTAADGYSGATALSQWAQGGAGALI-NNATGQTITWM       261
AKB88576.1                  -----------------------WTSADGFSGATPLSQWSVNGSHSLV-NTVSGQPITWM       261
WP_011020707.1              --------------------------------------F-------------S-------       150
AKE29920.1                  ----------------------WKSPADVVTAATPLALFKEHGVF----TPYWDL-----       177
AFA48979.1                  ------------------------PLTDTVTTATPLALLKAGETGSM--PSTLDL-----       175
SQB91608.1                  --------------------------LDGVTTATPLAILKGNEATGAAAPDLMSV-----       174
CAC03727.1                  GWLEGLRITFAGGAL-----------ADGLSGATALGHLQTELTLGHSAAQILDGHFALL       191
AZP58873.1                  DSLREFLG--------------LATRPDAWAHATALDVLKTDR--SLTVDELFAG-----       191
EFF12894.1                  GFIDAIQVIFSGHTASGGDMNTLRLGIDGISQATPLDTFKTSVRAGHSVEQIMQY-----       208
KPF39873.1                  DMLDTLRMIFTGHTASGGDMTLLRIGIDGISQATPLDTFKTSLRAGHSVEQIMQY-----       208

GAD71712.1                  DAFVGNLPGSMGEVSTLLIALTGLVLIFMKIASWRIVAGVLVGLIGASLLLNAIG---SD       317
AAX87224.1                  DAFVGNLPGSMGEVSTLAILIGGAVIVFTRIAAWRIIAGVMIGMIATSTLFNLIG---SE       315
AKB05379.1                  DAFIGNIPGSIGEVSTLALMIGAAFIVYMGIASWRIIGGVMIGMILLSTLFNVIG---SD       318
AKB88576.1                  DAFLGYIPGSIGEVSTLMILIGGAIIIFGRVASWRIVAGVMIGMIATAYLFNWIG---ST       318
WP_011020707.1              DLILENGAGFLTDVSPILVLLAGVILILVKYIEWRIPLSYLLTTVILALV---------L       201
AKE29920.1                  F--IGKVGGSLGETSALLLIIGFIYLLLRKRVKIFIPVSYIGTVLVFSSIAYLMN--PRY       233
```

```
AFA48979.1                  FTGLNGVYGCIGEISALALLIGGLYLIYKGIISWRIPTIYLLTIAIFALL----V--G--        227
SQB91608.1                  F--IGHVGGCIGETSALALLIGGAYLFYKHIIDWRIPVSFIGTTFIFTAI----A--GRG        226
CAC03727.1                  PAFLGYSGGSLGETSELLILLGGLWLLALRIIHWEIPLGMLLTVGALAALANQI-DPQVH        250
AZP58873.1                  NPAFGHLGGAGSEWVNLAFLLGGLFLLWRRLFTWHAPLGMLAGLFAMSLLFWNGSGSDSH        251
EFF12894.1                  PIYSGILAGAGWQWVNLAWLAGGVWLLWQKAIRWHIPFSFLVTLALCATLGWLFS-PETL        267
KPF39873.1                  PIYSGALAGVGWQWVNLAWLVGGVFLLWQKAIRWHIPVSFLLTLALCAALGWLFS-PATL        267
                                    *   :   :        :.             :      : :

GAD71712.1                  SNPMFSMPFYWHFVIGGVAFGTFFMATDPVSAAFTNNGKWAYGLLIGVMTIFIRVLNPAY        377
AAX87224.1                  TNPMFSMPWHWHFVLGGFALGMVFMATDPVSASFTNTGKWWYGALIGVMAVLIRTVNPAY        375
AKB05379.1                  TNAMFNMPWHWHLVLGGFAFGMFFMATDPVSASFTNSGKWAYGILIGVMCVLIRVVNPAY        378
AKB88576.1                  TNPLFAMPWYWHLVLGGFAFGMIFMATDPVSASFTNKGKWWYGGLIGVMCILIRVANPAY        378
WP_011020707.1              GDPL------AYVVSGTFLLGVFFIATETVTSPVTQNGRIVYGILCGFLTVIYGYFSGNY        255
AKE29920.1                  GDPL------FHLLSGGLMLGALFMATDMVTSPITAKGQVIFGIGCGVLTMAIRLF-GAY        286
AFA48979.1                  QDPI------VHMVSGGVMLGAFFMATDYASSPVTAKGQIIYAIGCGLITMIIRLY-GGY        280
SQB91608.1                  SSPV------YELFAGGLMLGAIFMATDYATSPITPLGRIIFGVGCGVITSLIRIF-GGY        279
CAC03727.1                  GGGL------FHLTSGGLLLGALFIATDPVTSPISRSGRLIFAIGCGALVFVIRSW-GNF        303
AZP58873.1                  GSPL------FHLFSGATMLGAFFIVTDPVSGATSNRGRLVFGLGVGVLTYVIRAW-GGY        304
EFF12894.1                  ASPQ------IHLLSGATMLGAFFILTDPVTASTTNRGRLIFGALAGLLVWLIRSF-GGY        320
KPF39873.1                  ASPQ------LHLLSGATMLGAFFILTDPVTASTTNRGRLIFGALAGVLVWLIRSF-GGY        320
                     .                  .  *   :* .*: *: .:.  :  *:  :.   * :          :

GAD71712.1                  PEGIMLAILFANLFAPLFDYLVKESNIKRRMKRARPS--------------        414
AAX87224.1                  PEGMMLAILFANLFAPIFDYIVVQANIKRRRARTNG---------------        411
AKB05379.1                  PEGMMLAILFANLFAPLFDHVVVERNIKRRLARYGKQ--------------        415
AKB88576.1                  PEGMMLAILFANLFAPLFDYVVVQANIKRRKARGE----------------        413
WP_011020707.1              VWGTLYALLLSNAVAPFIELKTLPKPMGGVANE------------------        288
AKE29920.1                  PEGVSFSILFMNALVPLIDRYTRPRIFGEVKK-------------------        318
AFA48979.1                  PEGCSYSILLMNVATPLIERFTKERIYGVTKIKKEAKA-------------        318
SQB91608.1                  PEGVSYSILVMNLFVPLIERWTAPKIFGKVK--------------------        310
CAC03727.1                  PEAVAFAVLLMNALVPLIDRVCRPRAYGRNARGKPLVAAKWTRQVKEVDKV        354
AZP58873.1                  PDGVAFAVLLMNLAAPTIDYYTRPRTYGHRKAERGFKAGD-----------        344
EFF12894.1                  PDGVAFAVLLANITVPLIDYYTRPRVYGHRKG-------------------        352
KPF39873.1                  PDGVAFAVLLANITVPLIDYYTRPRVYGHRKG-------------------        352
                              .   ::*. *  .* ::
```

e

```
TWU82118.1 H. influenza     -----------MSGKTSYKDLLLAPIAKNNPIALQILGICSALAVTTKLETAFVMAIAVT        49
AKB89713.1 Y. pestis       -----------MADSKEIKRVLLSPLFDNNPIALQILGVCSALAVTTKLETALVMTLAVT        49
ATD25239.1 V. cholerae     -----------MSSAKELKKSVLAPVLDNNPIALQVLGVCSALAVTTKLETAFVMTLAVM        49
GAD71946.1 V. alginolyticus -----------MSSAQNVKKSILAPVLDNNPIALQVLGVCSALAVTTKLETAFVMTLAVT        49
AAM04104.1 M. acetivorans  -------MYPHRRADMNPISEFIRGITKDNPTFGLVLGLCPTLAVTTSVENGIGMAMGTL        53
AGL49171.1 T. maritima     ---------------MSRLRELTKGIIKENPTYVQVLGMCPTLAVTTSAINGLGMGLATT        45
ACR23745.1 A. woodii       ---------------MNFMKNLTRGIIRENPTFVLVLGMCPTLAVTTSAINGMGMGLATM        45
SQB91610.1 C. tetanomorphum --------------MGVVSERLYNGIVKENATFVQVLGMCPTLAVTTSAINGIGMGLSAT        46
CAC03729.1 P. stutzeri     MSSQCGSADVTAPKPKGLFNYFSSALWDYNVALVQMLALCPALAVTTTATNGLGMGLATT        60
AZP58871.1 P. aeruginosa   ------------MSEQDFREIARNGLWRNNPGLVQLLGLCPLLGTSNSTVNALGLGLATM        48
CAD6013372.1 E. coli      --------------MSEIKDVIVQGLWKNNSALVQLLGLCPLLAVTSTATNALGLGLATT        46
CCW74136.1 S. enterica     --------------MSEIKDIVVQGLWKNNSALVQLLGLCPLLAVTSTATNALGLGLATT        46
                                                     :   *     :*.:*  *..:..  ..: : :..

TWU82118.1                  LVTGLSNLFVSLIRNYIPNSIRIIVQLAIIASLVIVVDQILKAYAYGLSKQLSVFVGLII        109
AKB89713.1                  LVTAFSSFFISLIRNHIPNSVRIIVQMVIIASLVIVVDQVLRAYAYEISKQLSVFVGLII        109
ATD25239.1                  FVTALSNFFVSLIRNHIPNSVRIIVQMAIIASLVIVVDQILKAYLYDISKQLSVFVGLII        109
GAD71946.1                  FVTALSNFSVSLIRNHIPNSVRIIVQMAIIASLVIVVDQVLKAYLYDISKQLSVFVGLII        109
AAM04104.1                  FVLVGSNMMVSAIRKGIPGTVRLPVEIIVIATFVTIVDMVMEAFTPDLYTSLGVFIPLIV        113
AGL49171.1                  AVLTMSNVVISLIRKIVPDKIRIPIFIVVIASFVTMIDLLMHGFAYDLWKTLGLFIPLIV        105
ACR23745.1                  LVLIGSNVAISALRKVIPDNIRIPAFVVVIASFVTIVGMLMKAYVPALDAALGIFIPLIV        105
SQB91610.1                  VVLIGSNVVISLLKKVIPDEIRIPAYITVIATLVTVLQFLLQAYLPDLNKSLGIFIPLIV        106
CAC03729.1                  LVLMITNAIISALRHSISPAVRNPLMIGIIAGVVTLIDMAINAWMHELYKVLGLFIALIV        120
AZP58871.1                  LVLACSNAAVSLVRGAVSEAIRLPAFVMIIAVLTTCIELLMQAWTYELYQVLGIFIPLIT        108
CAD6013372.1                LVLTLTNLTISTLRHWTPAEIRIPIYVMIIASVVSAVQMLINAYAFGLYQSLGIFIPLIV        106
CCW74136.1                  LVLTLTNLTVSALRRWTPAEIRIPIYVMIIASVVSAVQMLINAYAFGLYQSLGIFIPLIV        106
                             *   :.  :* ::      :*    : :** ..  :   :..:   :   *.:*: **

TWU82118.1                  TNCIVMGRAEAFAMKSPPVESFVDGIG-NGLGYGSMLIIVAFFRELIGSGKLFG------        162
AKB89713.1                  TNCIVMGRAEAYAMKSPPIESFMDGIG-NGLGYGVILVLVGFVRELVGSGKLFG------        162
ATD25239.1                  TNCIVMGRAEAFAMKSEPIPSFIDGIG-NGLGYGFVLMTVGFFRELLGSGKLFG------        162
GAD71946.1                  TNCIVMGRAEAFAMKSAPVPSLIDGIG-NGLGYGFVLITVGFFRELFGSGKLFG------        162
AAM04104.1                  VNCIVIGRAEAYALKNGVFYSIIDALG-EGTGFLLVLILIGGIRELLGTGIIDP------        166
AGL49171.1                  VNCIIMGRAESFASKHGVLDSMLDGLG-VGLGFTGSLVLLGSVRELFGNGTIFG------        158
ACR23745.1                  VNCIILARAEAFAFSNGIADSFADAVG-MGLGFTLALTILGSIREILGAGSIFG------        158
SQB91610.1                  VNCIILGRAEAYANKNSVGASFFDGLG-MGLGFTVSLAALGIIREFLGTGKVFG------        159
CAC03729.1                  TNCAVLGRAESFCSRNPVLPSILDGRRAWASGFTWVLVVIGGIREILGQRARCSPRPRLL        180
AZP58871.1                  TNCVILGRAEAFAAKNGVLRASFDGLL-MGLGFALVLLVLGGLRELLGQGTLLA-DMHLL        166
CAD6013372.1                TNCIVVGRAEAFAAKKGPALSALDGFS-IGMGATCAMFVLGSLREIIGNGTLFD-GADAL        164
CCW74136.1                  TNCIVVGRAEAFAAKKGPWLSALDGFS-IGMGATGAMFVLGSLREILGNGTLFD-GADSL        164
                            .** ::.***::.       :  *.    . *    :  :. .**:.*
```

```
TWU82118.1                    ----MTIFETIQNGGWYQANGLFLLAPSAFFIIGFVIWGLRTWKPEQQEK----------          208
AKB89713.1                    ----VTVLETVQNGGWYLPNGLFLLAPSAFFIIGLLIWGLRTLKPAQIEKE---------          209
ATD25239.1                    ----LEVLPLISNGGWYQPNGLMLLAPSAFFLIGFMIWAIRTFKPEQVEAKE--------          210
GAD71946.1                    ----LEVLPLVSNGGWYQPNGLMLLAPSAFFLIGFLIWVIRILKPEQVEAKE--------          210
AAM04104.1                    ----FGMT-LINLSGVINPAMFMTMSPGAFLTIAVLMTIVNYRRQQKAAKGG--------          213
AGL49171.1                    ----YKVW-----EL---KIFLEILPPGAYITLGLLSALFTYIGIRRKKRGEAK------          200
ACR23745.1                    ----FSLF-----GAAYEPVLLMILPPGAFLTLGLLIGLINWKTKKA-------------          196
SQB91610.1                    ----AQIT-----PDAFQPALIMILAPGGFFTLGILMAILNQRKLKKAK---AK------          201
CAC03729.1                    LGEHFRWL-EITVLPGFQGILLAILPPGAFIVLGFVLAFKRVVDRRRAERRIRTHGELVV          239
AZP58871.1                    FGPAAADW-KIQPFPQYQGFLLAILPPGAFIMLGLLIALKNRIDESLAERAKVQAGDVPA          225
CAD6013372.1                  LGSWAKVL-RVEIFHTDSPFLLAMLPPGAFIGLGLMLAGKYLIDERMKKRRAEAAAERAL          223
CCW74136.1                    LGGWAKVL-RVEIFHTDSPFLLAMLPPGAFIGLGLMLAVKYLIDEKMKKRRAETA-PSAV          222
                                          :  : *..:: :..:

TWU82118.1                    -------------      208
AKB89713.1                    -------------      209
ATD25239.1                    -------------      210
GAD71946.1                    -------------      210
AAM04104.1                    -------------      213
AGL49171.1                    -------------      200
ACR23745.1                    -------------      196
SQB91610.1                    -------------      201
CAC03729.1                    LQ-----------      241
AZP58871.1                    TQRQRVRVTGVIE      238
CAD6013372.1                  PNGETGNV-----      231
CCW74136.1                    PAGETGKV-----      230
```

f

```
AAX87225.1  H. influenza      ----------------------MAKFNKDSVGGTILVVLLLSLVCSIIVAGSAVMLKPAQ          38
AKB87827.1  Y. pestis         -------------------MASDKPRNNDSIGKTLLVVVILCLVCSVVVAGAAVGLKAKQ          41
KKP21788.1  V. cholerae        -----------------------------------------MCSIIVSAAAVGLRDKQ          17
GAD71945.1 V. alginolyticus  ------------------------MASNNDSIKKTLGVVIGLSLVCSIIVSTAAVGLRDKQ          37
CAC03728.1  P. stutzeri       MNELTQTPPVADGNEPPFTRPGLVETWRERVSYQALSLGLVCALVAVALLLGNQLTHQRI          60
AZP58872.1  P. aeruginosa     ----------------------MDAATRRSMLRNALLLGLFALVGVGLVALVQQFTEARI          38
CAD6006652.1 E. coli         --------------------------MLKTIRKHGITLALFAAGSTGLTAAINQMTKTTI          34
CCW74137.1  S. enterica       --------------------------MLKTIRKHGITLALFAAGSTGLTAVINQMTKSTI          34
AKE29921.1  T. maritima       ---------------------------MKDILKTGLILMVFTAISGLFLGLVYVGVKGKI          33
sp|Q8TSY2.1 M. acetivorans -------------------------MSDSKEITKVIVTMVVISAVAAALLALTYTPTQAQL          36
ACR23744.1  A. woodii         -----------------METKEKVQIDWKVVFKLGLILFVISAVAACALALTNYVTAGTI          43
SQB91609.1 C.tetanomorphum -------------------------MKKVSSFKLGMVLLLIAAVCGLILGGVNQVTAEPI          35

AAX87225.1                    EEQKLLDKQKNILNVAGLLQAN---TNVKETYAKFIEPRFVDLATGEYTQQADDS-----          90
AKB87827.1                    QEQRLLDKQRNILAVAGLLQPRMLAEEVQQAFATRIEPRLLDLQSGEFLKQDPATFDRSQ          101
KKP21788.1                    KENAALDKQSKILQVAGIEAKG--SKQIVELFNKSIEPRLVDFNTGDFVEGDAANYDQRK          75
GAD71945.1                    KANAVLDKQSKIVEVAGIDANG--K-KVPELFAEYIEPRLVDLETGNFTEGNASTYDQRE          94
CAC03728.1                    VDAERQDRLAVLRQVLP------------------------QALYDNDPLADAFNVED--          94
AZP58872.1                    AEAQREARGRALLELLP------------------------PGSYDNHPLDSQVPTFA--          72
CAD6006652.1                  AEQASLQQKALFDQVLP------------------------AERYNNALAQSCYLVTA--          68
CCW74137.1                    HEQALQQQHALFDQVLP------------------------PDRYNNNLQESCYLVDA--          68
AKE29921.1                    QEADNAAKLSAIKFVLK------------------------DPLTGDYL-VDEKEIEE--          66
sp|Q8TSY2.1                   KLLQAEQQKEAMKEILP------------------------QA--ADFEPVTGSEVDD--          68
ACR23744.1                    EEMNVQTNTVARQEVLP------------------------KA--ADFEAVPAKDVEK--          75
SQB91609.1                    AIQNKKTLDEANKAILP------------------------EA--SEFAEKT--------          61
                                              :                                 :

AAX87225.1                    -------QQAIPADADKARIRSRSKTTEVYLVKDEQGQTQQVI--------LPIYGTG--          133
AKB87827.1                    ALRDNQMSIALTPAQDIAGIRRRANVVEIYLVRGDGGQINKVI--------LPIYGSG--          151
KKP21788.1                    AAKEASESIKLTAEQDKAKIQRRANVGVVYLVK-DGDKTSKVI--------LPVHGNG--          124
GAD71945.1                    ASKDAERSIALTPEEDVADIRRRANTAVVYLVK-DQDEVQKVI--------LPMHGKG--          143
CAC03728.1                    --------------AEL----G---LIEVYPARRAGQLTATAFQI---------STVGYG          124
AZP58872.1                    --------------PKLLGLDA---PRPAYVARLHGQASAVILQAS--------APDGYS          107
CAD6006652.1                  --------------PEL-GK-G---EHRVYIAKQDDKPVAAVLEAT--------APDGYS          101
CCW74137.1                    --------------PAL-GK-G---THRVFIARKDDKPVAAIIEAT--------APDGYS          101
AKE29921.1                    ----------I---VKKTGIET-VVLKEYKEGVVLGPLYEFVTKDGRNAYVLSGYAPGFG          112
sp|Q8TSY2.1                   --------------------DGNPVVLYYKGVDSSGNVVGYVVERN--------Q-VGAQ          99
ACR23744.1                    ----------I---ASEIGMEKPEELLEVYIGKSNGEVVGYTVKTG--------PTSGYA          114
SQB91609.1                    ------------------DIKGEGIVLGVTEGKSGSDLKGYTIKVA--------P-KGYA          94
                                                                    *
```

```
AAX87225.1              -LWSVMYGLVSVQPDGNTINGITYYQHGETPGLGGEIEN--PNWASLFKGKKLFDEQHQP    190
AKB87827.1              -LWSMMYAFVAIDTDGKTVRGITYYDHGETPGLGGEIEN--PIWRNQWIGKRLFDDQGQP    208
KKP21788.1              -LWSMMYAFVAVETDGNTVSGLTYYEQGETPGLGGEVEN--PAWRAQWVGKKLFDENHKP    181
GAD71945.1              -LWSMMYAFVAVETDGNTVSAITYYEQGETPGLGGEVEN--PSWRDQFIGKKLYNEDHQP    200
CAC03728.1              GPIVQFIALD---SE-GRILGVRVLSHKETPGLADKIEVTRSDWIKAFDGLSLASTPLDQ    180
AZP58872.1              GAIQLLVGVT---AQ-GRLLGVRVVAHKETPGLGDRIELAKSPWVHGFDGKSLGDPADAG    163
CAD6006652.1            GAIQLLVGAD---FN-GTVLGTRVTEHHETPGLGDKIELRLSDWITHFAGKKISGADDAH    157
CCW74137.1              GAIQLIVGAD---FN-GTVLGTRVTEHHETPGLGDKIERRLSDWITHFSGKTISGENDTH    157
AKE29921.1              GNVTVVACFIKTEDGFMLNSVRVIDYSQETPGLGAKIGE--ESIQRRFFPVPPEGLKNGL    170
sp|Q8TSY2.1             GMIQLLAGIS---SDFSTITGFQVMKHSETPGLGALITT--PEFQGQFVDLPVADTS---    151
ACR23744.1              GEVQVLTGIS---AD-GVITGITIIKSNETPGLGAKASG--V-WNDQFTGKSAKEEL---    164
SQB91609.1              GAIEMMVGVS---TE-GKVTGIKILNHAETPGLGANATD--PKFSGQYANKPAK-EL---    144
                             .                      *****.             :

AAX87225.1              ----AIRIVKG----QAPQ-DEHSIDGLSGATLTGNGVQGTFNYWFSKDGFGPYLEKLHS    241
AKB87827.1              ----AIRIVKG----RAPANDPHAVDGLSGATLTSNGVQNSFNFWLGENGFGPFLKKVRE    260
KKP21788.1              ----AIKIVKG----GAPQGSEHGVDGLSGATLTSNGVQNTFDFWLGDMGFGPFLTKVRD    233
GAD71945.1              ----AIKVVKG----GAPQGSEHGVDGLSGATLTSNGVQHTFDFWLGDMGFGPFLAKVRD    252
CAC03728.1              ---------------WAVKKDGGQFDQFAGATITPRAIVKGVLRAL------EFQARQST    219
AZP58872.1              ---------------WAVKKDGGTFDQFAGATVTPRAVVRAVHKAL------RYFDANRE    202
CAD6006652.1            ---------------WAVKKDGGDFDQFTGATITPRAVVNAVKRAG------LYAQTLPA    196
CCW74137.1              ---------------WAVKKDGGDFDQFTGATITPRAVVNAVKRAG------LYAESLPA    196
AKE29921.1              RVDKDAGLPKGSPEELKKQGIVKVSDVMTGATITPRAVVTALNLMY------RYLEEVSK    224
sp|Q8TSY2.1             L------------TK-----NGGQVDAISGATISSQAVVDALHSAV------DYVSAQEG    188
ACR23744.1              VV------VKGTTKE-----GSNEIQAITGSTITSKAVTSGVNMSI------QVYQNLSK    207
SQB91609.1              KV------VKG-AAS-----GEDEIVAITGATITSKAVTLGVNEAI------KFYDTKLK    186
                                                   ::*:*:: ..:   .


AAX87225.1              GAN---------       244
AKB87827.1              GALKNG------       266
KKP21788.1              GGLN--------       237
GAD71945.1              GELN--------       256
CAC03728.1              AQSNQETRP---       228
AZP58872.1              RLLAPEEAAGHE       214
CAD6006652.1            QLSQ-LPACGE-       206
CCW74137.1              QLPH-LTACGE-       206
AKE29921.1              ------------       224
sp|Q8TSY2.1             ------------       188
ACR23744.1              ------------       207
SQB91609.1              GGK---------       189
```

Supplementary Fig. 5: **Sequence alignment of Nqr from *Vibrio cholerae, Vibrio alginolyticus, Yersinia pestis* as well as *Haemophilus influenza, and Rnf* from *Acetobacterium woodii, Escherichia coli, Thermotoga maritima, Salmonella enterica, Methanosarcina acetivorans, Pseudomonas stutzeri, Pseudomonas aeruginosa and C. tetanomorphum.*** Nqr subunits are highlighted in gray. **a** RnfA, Cys25 and Cys113 are highlighted in red. **b** RnfB. [4Fe-4S]$_{\mathrm{RnfB1\text{-}6}}$ clusters are highlighted in red, yellow, gray, green, blue and magenta based on the Rnf structure of *C. tetanomorphum*. The [4Fe-4S] cluster content varies in Rnfs from different microorganisms. *T. maritima* RnfB carries only the first [4Fe-4S] cluster of FdI, *C. tetanomorphum*, in addition, the five [4Fe-4S] clusters of FdII+III and *A. woodii* RnfB even two further [4Fe-4S] clusters in a new FdIV segment [12]. *E. coli* Rnf host besides the FdI segment (red) only the FdII unit with one of the two [4Fe-4S] clusters (gray) differently ligated compared with *C. tetanomorphum* FdII. **c** RnfC. The two [4Fe-4S] clusters were marked in yellow and green. **d** RnfD. Residues of constriction were highlighted in green, Asp248 and Asn124 in magenta, those contacting FMN in yellow and Thr153 in blue. **e** RnfE. Cys26 and Cys109 are highlighted in red. **f** RnfG. Thr164 is shown in blue. According to the genome sequence [13] the Rnf complex has a molecular mass of 169.253 kDa (RnfA 20.584 kDa; RnfB 27.923 kDa, RnfC 47.103 kDa, RnfD 32.968 kDa, RnfG 19.478 kDa, RnfE 21.197 kDa).

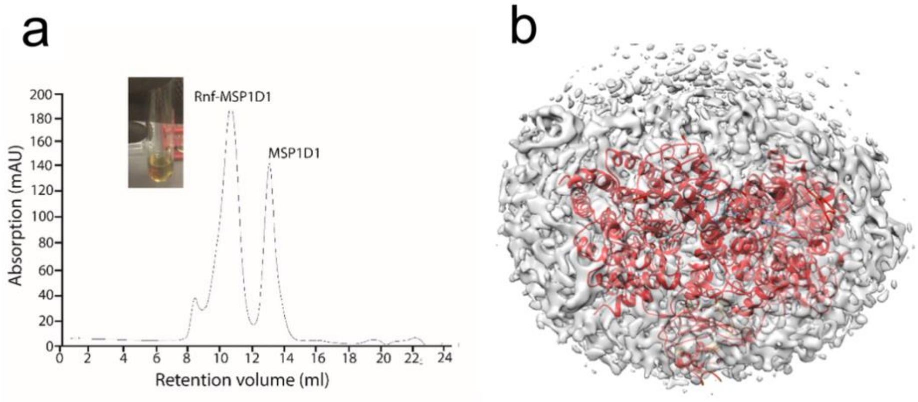

Supplementary Fig. 6: **Rnf in nanodiscs. a** Reconstitution of Rnf into nanodiscs MSP1D1[14,15] replacing the lipid DMPC. Lipid-filled and Rnf-filled nanodiscs could be separated by gelfiltration (Superdex 200 HR 10/300 GL) **b** Cryo-EM map (top view) indicating the disordered but visible nanodisc belt around a Rnf model (red ribbon).

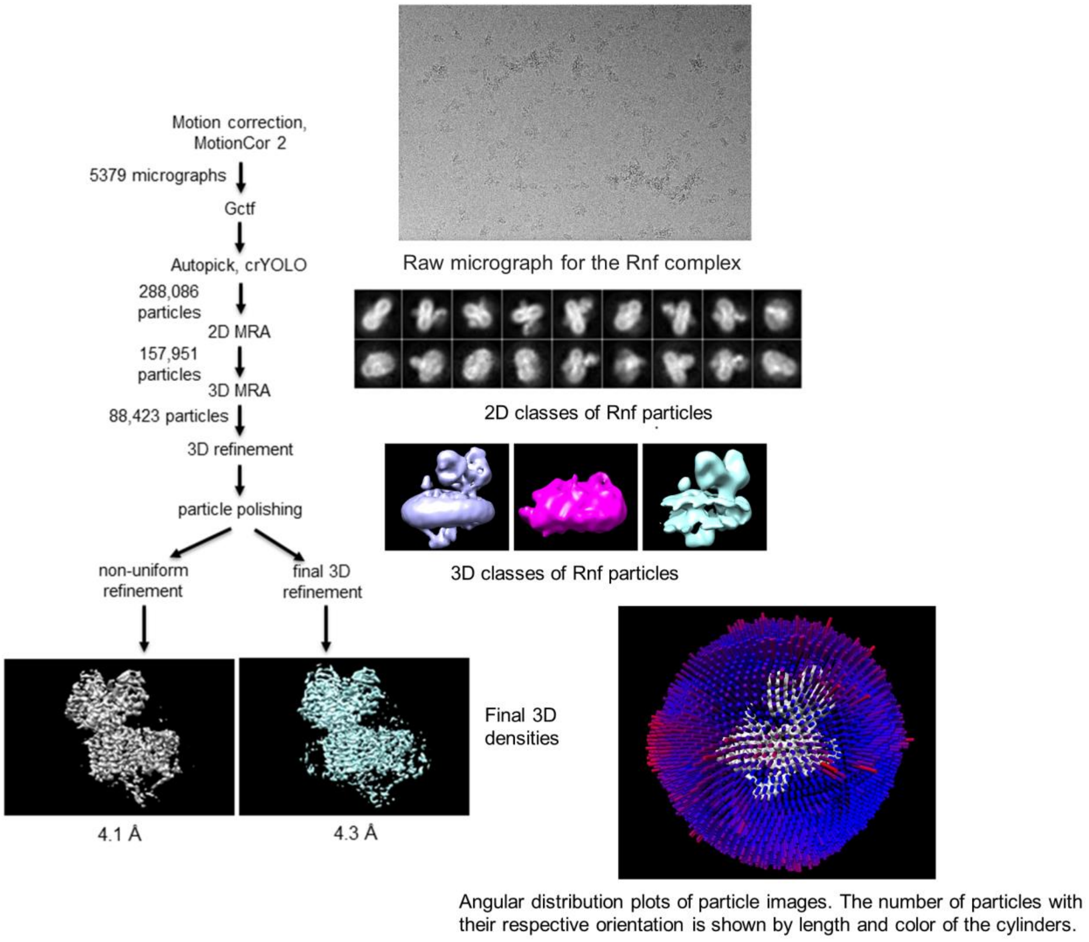

Supplementary Fig. 7: Workflow of Rnf structure determination using Relion [16] for data processing. Data collection was done on a Titan Krios EM at 300 kV equipped with a Gatan K3 detector with a pixel size of 0.837Å at 105000x magnification. Data processing is a multi-step process described in the Method section. The figure also shows the major 2D and 3D classes based on multiple reference alignment (MRA) and the angular distribution of the particles in the micrographs that represents an indicator for the quality of 3D refinement.

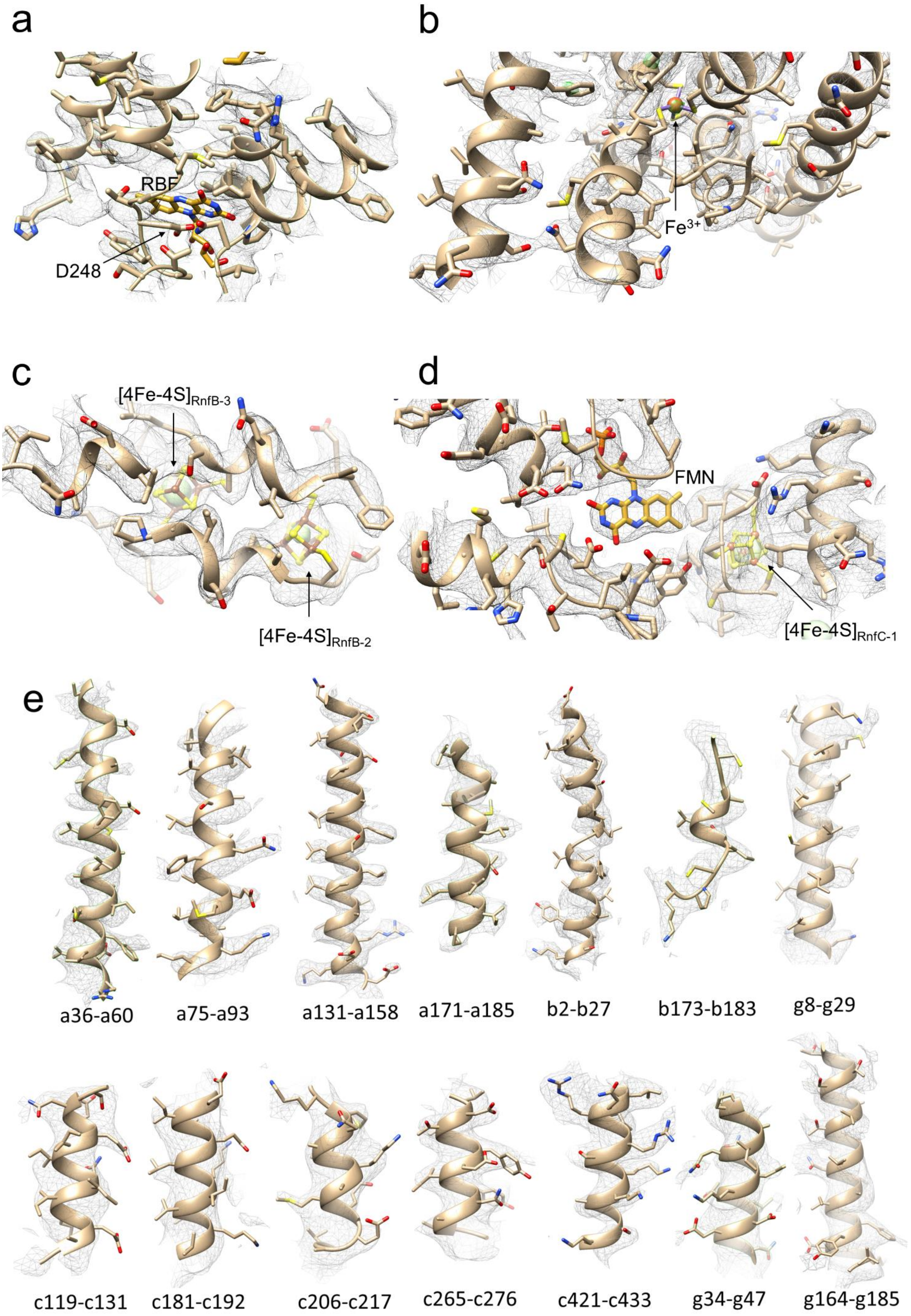
a
RBF
D248
b
Fe3+
c
[4Fe-4S]RnfB-3
[4Fe-4S]RnfB-2
d
FMN
[4Fe-4S]RnfC-1
e
a36-a60
a75-a93
a131-a158
a171-a185
b2-b27
b173-b183
g8-g29
c119-c131
c181-c192
c206-c217
c265-c276
c421-c433
g34-g47
g164-g185

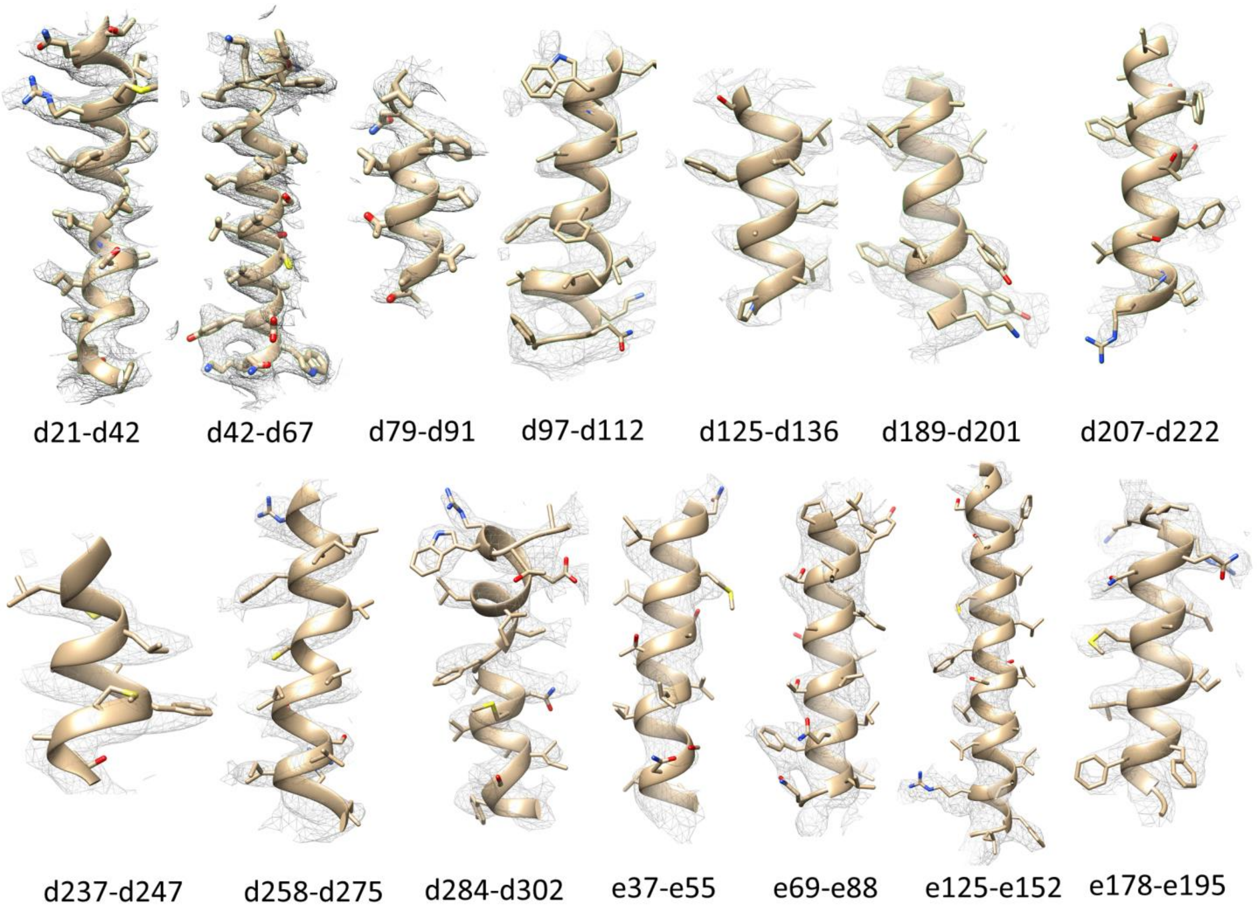

Supplementary Fig. 8: The density map at 4.27 Å resolution. Main chain modeling was feasible except for residues 31-95 of RnfB. The profile of some characteristic side chains was sufficient for reliable interpretation, which allowed the approximate assignment of those in between. Solely, the side chains of the N-terminal helix of RnfB were set to zero. Regions **a** around the riboflavin (RBF) and Asp248 of RnfD, **b** around the potential Fe ion between RnfA and RnfE, **c** around the $[4Fe\text{-}4S]_{RnfB\text{-}II}$ and $[4Fe\text{-}4S]_{RnfB\text{-}III}$ clusters and **d** around FMN of RnfC.. The main chain was drawn in the Ribbon style and the side chains and cofactors as sticks and spheres. Contour levels 1 and 2 are drawn as gray mesh and as green surface, respectively. **e** Density and model of all helices of the Rnf complex.

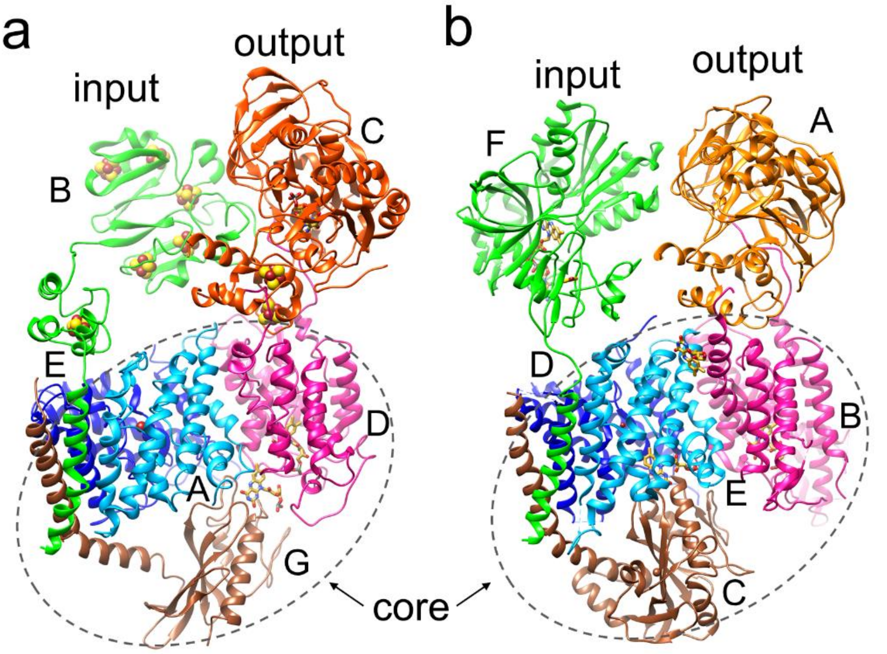

Supplementary Fig. 9: Comparison between the architecture of Rnf **a** and Nqr **b**. The core complexes RnfADEG and NqrEBDC are structurally and functionally highly related. RnfB/NqrF (green) and RnfC/NqrA (orange), the input and output modules in the Na^+ pumping direction, are spatially arranged in the same manner relative to the core complex implicating that the two NAD binding subunits RnfC and NqrF have to be attached to different sides. It is worth to note that the structurally related RnfC and NqrA perform different catalytic reactions.

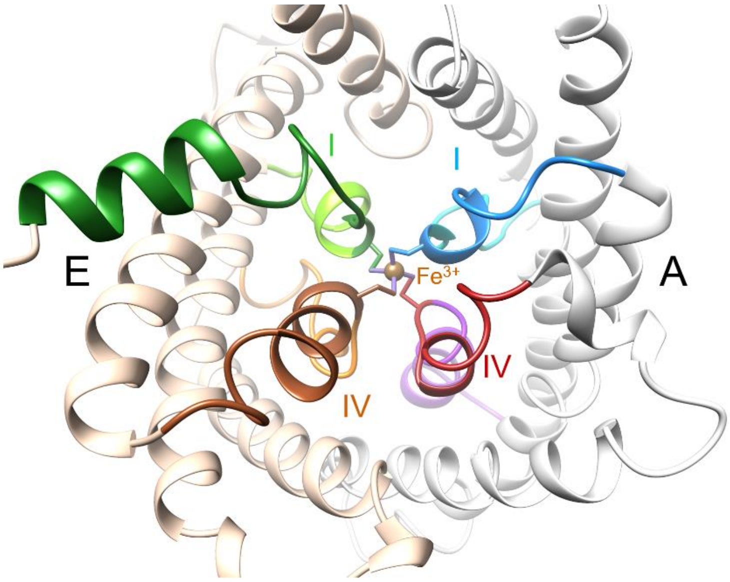

Supplementary Fig. 10: Hourglass-like architecture of RnfAE. Helices I and IV of RnfA and RnfE are kinked at the central Fe^{3+} binding site are extended outwards towards the extra- and intracellular monolayer forming an hourglass-like arrangement. Half-helices 13:24 and 25:33 of RnfA are drawn in sky blue and cyan; half-helices 103:113 and 114:128 of RnfA in dark red and purple; Half-helices 5:14 and 27:34 of RnfE are highlighted in dark green and green, half-helices 96:108 and 109:122 of RnfE in brown and orange.

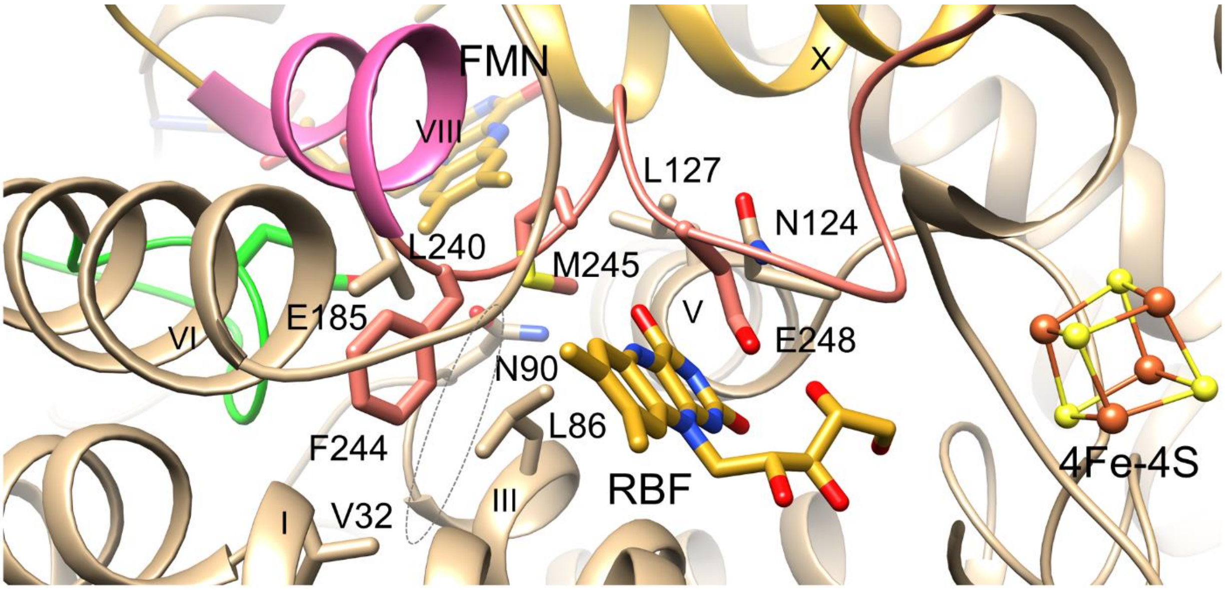

Supplementary Fig. 11: Detailed view of the region between FAD_{RnfD} and the $[4Fe\text{-}4S]_{RnfC\text{-}2}$ cluster in the closed-channel enzyme state. The key player is helix 237:246 (VIII) (purple) non-covalently joined with helices X (gold), VII and IX (the latter not shown). Loops that follow helix VIII and occlude the extracellular half-channel are drawn in salmon and green. In the main text the electron flow from Fd_{red} to NAD^+ coupled with an uphill Na^+ transport was already described in detail. In the reverse direction FAD_{RnfD} and the riboflavin (RBF) interspace is again the scene of the central coupling process. Reduction of RBF (from neutral semiquinone to reduced hydroquinone in Nqr[17]) might result in a repulsion between the negatively charged C-terminal end of helix VIII (purple) including Asp248 and the electron-rich reduced riboflavin that soften helix VIII. As a consequence, the cytoplasmic Na^+ binding site localized in this region is impaired while Na^+ can reach the hydrophobic barrier (dashed ellipse) from the extracellular half-channel because the occlusion loop (green) is postulated to be refolded into two helix windings when RnfG is attached to RnfAE (Fig. 5). Driven by the high extracellular concentration, Na^+ binding (assumed next to Asn90, Glu185 and the tip of the occlusion loop) induces a rearrangement of the already disengaged helix VIII together with several other helices including helix 284:293 (X) (gold) thereby adjusting the strained open-channel state. Concurrently, Na^+ diffuses through the widened hydrophobic barrier and perhaps through an opened cytoplasmic half-channel. Finally, FMN_{RnfD} accepts an electron from reduced riboflavin, which triggers a subsequent strained-to-relaxed state transition. In parallel, RnfG is rotated to RnfD and the occlusion loop (green) blocks the access to the constriction.

References

1. Buckel, W. & Thauer, R.K. Energy conservation via electron bifurcating ferredoxin reduction and proton/Na^+ translocating ferredoxin oxidation. *Biochim Biophys Acta* **1827**, 94-113 (2013).
2. Buckel, W. Energy conservation in fermentations of anaerobic bacteria. *Front Microbiol* **12**, 703525 (2021).
3. Demmer, J.K., Pal Chowdhury, N., Selmer, T., Ermler, U. & Buckel, W. The semiquinone swing in the bifurcating electron transferring flavoprotein/butyryl-CoA dehydrogenase complex from *Clostridium difficile*. *Nat Commun* **8**, 1577 (2017).
4. Boiangiu, C.D. et al. Sodium ion pumps and hydrogen production in glutamate fermenting anaerobic bacteria. *J Mol Microbiol Biotechnol* **10**, 105-19 (2005).
5. Suloway, C. et al. Automated molecular microscopy: the new Leginon system. *J Struct Biol* **151**, 41-60 (2005).
6. Grant, T., Rohou, A. & Grigorieff, N. cisTEM, user-friendly software for single-particle image processing. *Elife* **7**(2018).
7. Bertsova, Y.V. et al. Alternative pyrimidine biosynthesis protein ApbE is a flavin transferase catalyzing covalent attachment of FMN to a threonine residue in bacterial flavoproteins. *J Biol Chem* **288**, 14276-86 (2013).
8. Bertsova, Y.V. et al. Alternative pyrimidine biosynthesis protein ApbE is a flavin transferase catalyzing covalent attachment of FMN to a threonine residue in bacterial flavoproteins. *J Biol Chem* **288**, 14276-14286 (2013).
9. Hamann, N. et al. A cysteine-rich CCG domain contains a novel [4Fe-4S] cluster binding motif as deduced from studies with subunit B of heterodisulfide reductase from Methanothermobacter marburgensis. *Biochemistry* **46**, 12875-85 (2007).
10. Nakamura, M., Saeki, K. & Takahashi, Y. Hyperproduction of recombinant ferredoxins in escherichia coli by coexpression of the ORF1-ORF2-iscS-iscU-iscA-hscB-hs cA-fdx-ORF3 gene cluster. *J Biochem* **126**, 10-8 (1999).
11. Wang, S., Huang, H., Moll, J. & Thauer, R.K. $NADP^+$ reduction with reduced ferredoxin and $NADP^+$ reduction with NADH are coupled via an electron-bifurcating enzyme complex in *Clostridium kluyveri*. *J Bacteriol* **192**, 5115-23 (2010).
12. Kuhns, M., Trifunovic, D., Huber, H. & Müller, V. The Rnf complex is a Na^+ coupled respiratory enzyme in a fermenting bacterium, *Thermotoga maritima*. *Commun Biol* **3**, 431 (2020).
13. Gong, F. et al. Fermentation and genomic analysis of acetone-uncoupled butanol production by *Clostridium tetanomorphum*. *Appl Microbiol Biotechnol* **100**, 1523-1529 (2016).
14. Faas, R. et al. Time-course and degradation rate of membrane scaffold protein (MSP1D1) during recombinant production. *Biotechnol Rep (Amst)* **17**, 45-48 (2018).
15. Denisov, I.G. & Sligar, S.G. Nanodiscs in Membrane Biochemistry and Biophysics. *Chem Rev* **117**, 4669-4713 (2017).
16. Scheres, S.H. RELION: implementation of a Bayesian approach to cryo-EM structure determination. *J Struct Biol* **180**, 519-30 (2012).
17. Barquera, B., Ramirez-Silva, L., Morgan, J.E. & Nilges, M.J. A new flavin radical signal in the Na^+-pumping NADH:quinone oxidoreductase from *Vibrio cholerae*. An EPR/electron nuclear double resonance investigation of the role of the covalently bound flavins in subunits B and C. *J Biol Chem* **281**, 36482-91 (2006).

Source data file for supplementary Fig. 3a

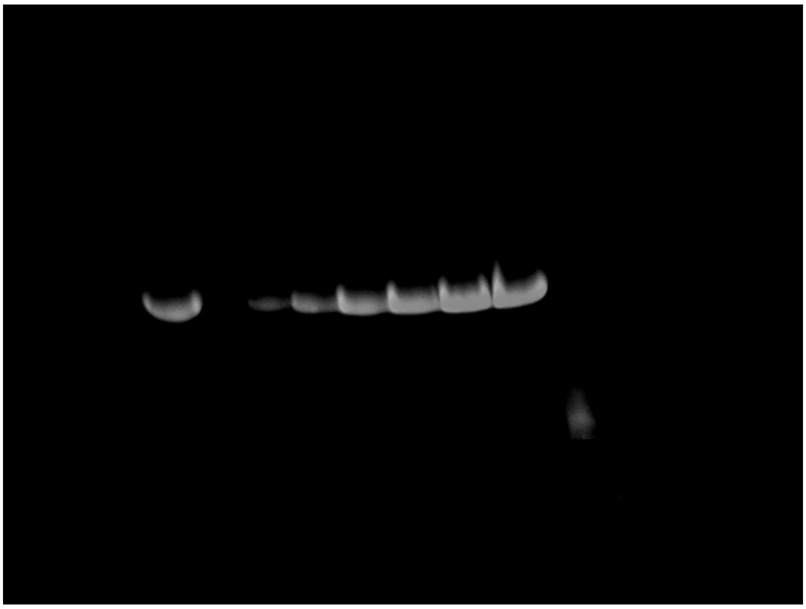

Supplement: Supplementary file 1 — Supplementary Information [file 41467_2022_34007_MOESM1_ESM.pdf]
