# Supplementary figures and images for: Purification and structural characterization of the Na+-translocating ferredoxin: NAD+ reductase (Rnf) complex of Clostridium tetanomorphum
Source: Nat Commun. 2022 Oct 23;13:6315. doi: 10.1038/s41467-022-34007-z (PMC9588780; doi:10.1038/s41467-022-34007-z)

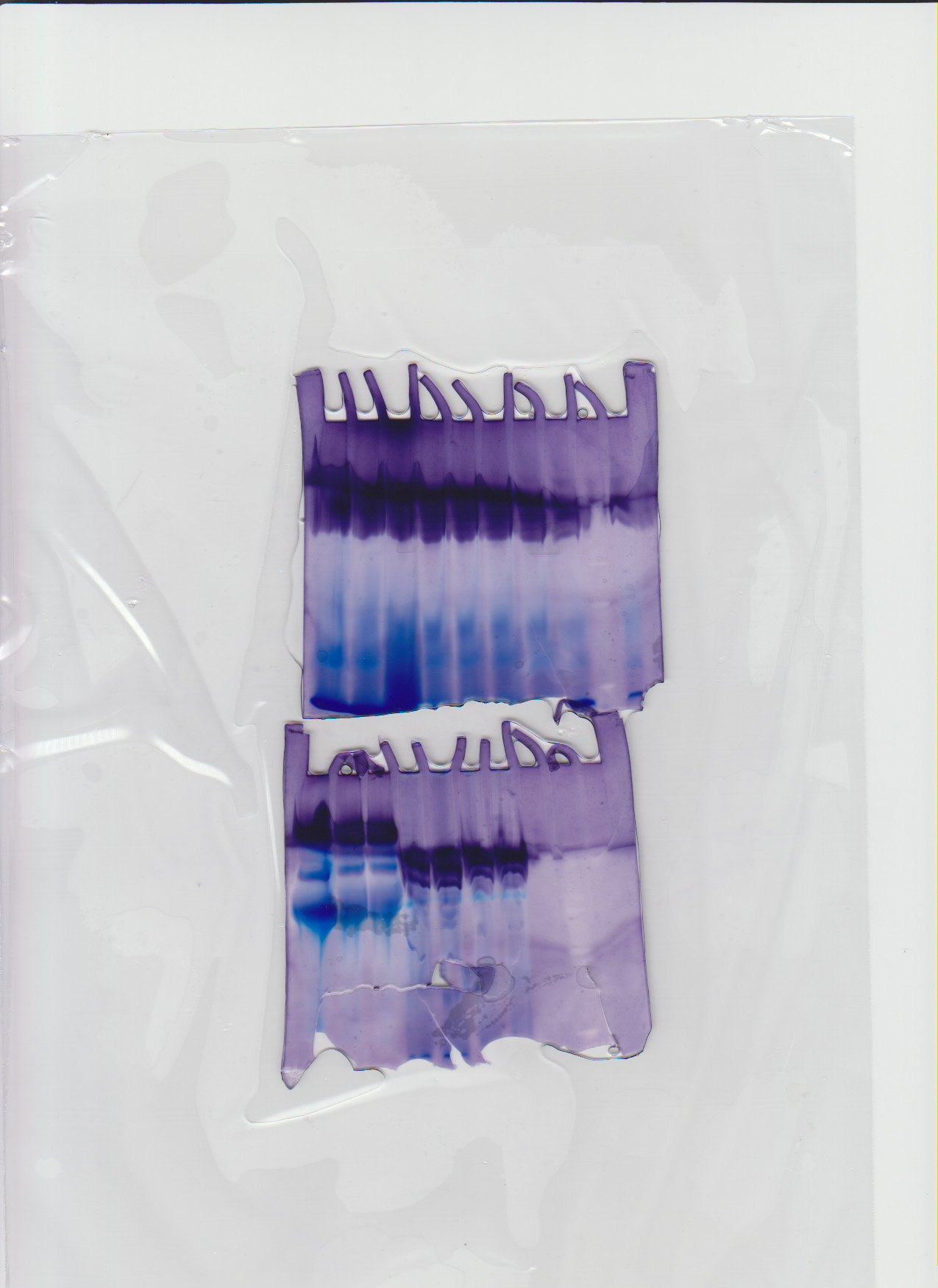

Supplement: Supplementary file 3 — Source Data [file 41467_2022_34007_MOESM3_ESM.zip › sourcefile1/aktivitatspage2.jpg]

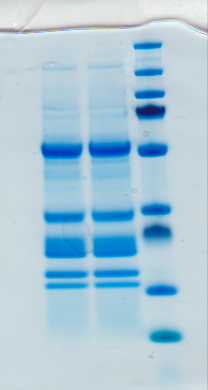

Supplement: Supplementary file 3 — Source Data [file 41467_2022_34007_MOESM3_ESM.zip › sourcefile1/rnf_reactivered.tif]

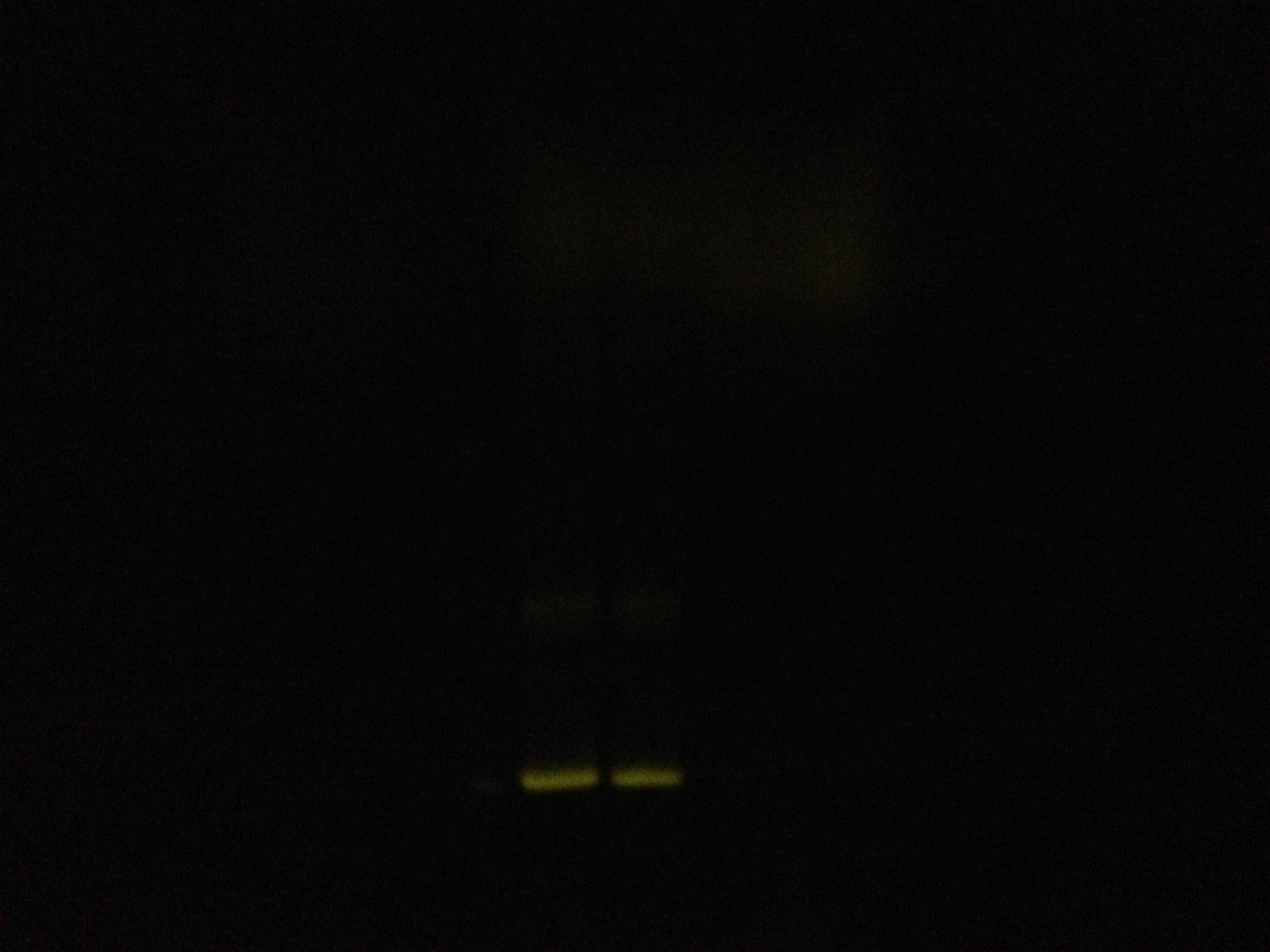

Supplement: Supplementary file 3 — Source Data [file 41467_2022_34007_MOESM3_ESM.zip › sourcefile1/rnf_gefi2 copy_fig1d.jpg]
